# Genome characterization of *Shewanella algae* in Hainan Province, China

**DOI:** 10.3389/fmicb.2024.1474871

**Published:** 2024-10-02

**Authors:** Licheng Wang, Shaojin Chen, Mei Xing, Lingzhi Dong, Huaxiong Zhu, Yujin Lin, Jinyi Li, Tuo Sun, Xiong Zhu, Xiaoxia Wang

**Affiliations:** ^1^Clinical & Central Laboratory of Sanya People’s Hospital, Sanya, China; ^2^Wenchang People’s Hospital, Wenchang, China

**Keywords:** *Shewanella algae*, genome characterization, Hainan Province, pangenome, virulence-associated genes, antibiotic resistance

## Abstract

*Shewanella algae* is an emerging marine zoonotic pathogen. In this study, we first reported the *Shewanella algae* infections in patients and animals in Hainan Province, China. Currently, there is still relatively little known about the whole-genome characteristics of *Shewanella algae* in most tropical regions, including in southern China. Here, we sequenced the 62 *Shewanella algae* strains isolated from Hainan Province and combined with the whole genomes sequences of 144 *Shewanella algae* genomes from public databases to analyze genomic features. Phylogenetic analysis revealed that *Shewanella algae* is widely distributed in the marine environments of both temperate and tropical countries, exhibiting close phylogenetic relationships with genomes isolated from patients, animals, and plants. Thereby confirming that exposure to marine environments is a risk factor for *Shewanella algae* infections. Average nucleotide identity analysis indicated that the clonally identical genomes could be isolated from patients with different sample types at different times. Pan-genome analysis identified a total of 21,909 genes, including 1,563 core genes, 8,292 strain-specific genes, and 12,054 accessory genes. Multiple putative virulence-associated genes were identified, encompassing 14 categories and 16 subcategories, with 171 distinct virulence factors. Three different plasmid replicon types were detected in 33 genomes. Eleven classes of antibiotic resistance genes and 352 integrons were identified. Antimicrobial susceptibility testing revealed a high resistance rate to imipenem and colistin among the strains studied, with 5 strains exhibiting multidrug resistance. However, they were all sensitive to amikacin, minocycline, and tigecycline. Our findings clarify the genomic characteristics and population structure of *Shewanella algae* in Hainan Province. The results offer insights into the genetic basis of pathogenicity in *Shewanella algae* and enhance our understanding of its global phylogeography.

## Introduction

The genus *Shewanella* is a group of gram-negative, facultative anaerobic motile bacilli, and mainly found in the marine environment. Since the first identification of *Shewanella putrefaciens* in 1931 (Yousfi et al., 2017), more than 70 species of the genus *Shewanella* have been described until now.^1^ Studies have shown that the main pathogenic bacteria of the genus *Shewanella* is *Shewanella algae* (*S. algae*), an emerging zoonotic opportunistic pathogen (Saticioglu et al., 2021; Ng et al., 2022; Yu et al., 2022). Cases of *S. algae* infections are currently being reported worldwide, particularly in countries with tropical, subtropical, and temperate climates where the infection rate of *S. algae* is high. At present, human infections in China have only been reported in the coastal regions of Shandong Province (Zhang et al., 2018), Taiwan (Liu et al., 2013), and Hong Kong (Ng et al., 2022).

*S. algae* infections occur mainly in coastal areas and are associated ingestion of contaminated seafood or exposure to coastal waters. The people are generally susceptible, especially those with underlying diseases or compromised immunity (Ng et al., 2022). The clinical presentation is like that of infections caused by *Vibrio vulnificus* or *Aeromonas* spp. The primary clinical syndromes, which usually appears after exposure to the marine environment, includes otitis media, ocular infections, and skin and soft tissue infections following local trauma. Other clinical manifestations also include hepatobiliary, respiratory, gastrointestinal, endocardial, and nervous system infections, as well as severe sepsis and bacteremia (Vignier et al., 2013; Yousfi et al., 2017; Yu et al., 2022). If treatment is not prompt and effective, mortality is high in compromised immunity patients or those with underlying medical conditions (Liu et al., 2013; Janda and Abbott, 2014; Torri et al., 2018; Ng et al., 2022; Yu et al., 2022).

The development of whole-genome sequencing has transformed many areas of research. Especially in the field of bacterial genomics, this technology can be used to clarify phylogenetic relationships among different species, providing a deeper understanding of the genomic properties of virulence and drug resistance in many important pathogens. It has now been confirmed that *S. algae* can produce a variety of virulence factors, such as tetrodotoxin, hemolysins, extracellular enzymes, and siderophores (Wang et al., 2013; Janda and Abbott, 2014; Rütschlin et al., 2018; Tseng et al., 2018). Further research has identified genes related to spoilage metabolism pathways (including trimethylamine metabolism, sulfur metabolism, cadaverine metabolism, biofilm formation, and serine protease production) as well as specific quorum sensing system genes in *S. algae* strains. This provides additional evidence for its role in food spoilage and pathogenicity to animals and humans (Li et al., 2020). In addition, several virulence factors and various mobile genetic elements were identified that contribute to the evolution and virulence of *S. algae* (Huang et al., 2022). Currently, there are no definite treatment options for *S. algae* infection. *S. algae* is a reservoir of drug-resistant genes that confer to first-and second-generation cephalosporins, penicillins, macrolides and carbapenems (Cimmino et al., 2016). However, the mechanisms of resistance are not fully understood. Little is known about the genomic characterization of *S. algae* from clinical sources. The acquisition of high-quality genomic data is becoming increasingly important for emerging pathogens and has become key to the study of pathogenesis and therapeutic interventions for emerging infectious diseases.

Hainan Province is surrounded by seas and located in the tropics. However, the studies of *S. algae* infection in Hainan Province, have never been reported in China and abroad. Recently, the *S. algae* infections were first detected in several patients and marine animals admitted to our hospital, and the *S. algae* infections have been seriously neglected in Hainan Province. However, there is so far no report on the genetic characterization of *S. algae* in Hainan Province. In this study, we sequenced the whole genome of *S. algae* strains collected from Hainan Province. Combined with the whole-genome sequences of 144 *S. algae* genomes from public databases, we performed genomic characteristics analysis to explore the population structure, relationship with global strains, virulence and antibiotic resistance factors, with the aim of improving the understanding of this emerging pathogen.

## Methods

### Strain information and genomic sequencing

In total, 206 *S. algae* genomes were included in this research, comprising 62 recently sequenced strains isolated from various source in Hainan Province and 144 genomes from the National Center for Biotechnology Information (NCBI) Genome database (http://www.ncbi.nlm.nih.gov/Genomes/, specifically selecting genomes with fewer than 200 contigs). Among them, 105 genomes were obtained from clinical specimens, 43 genomes from animals, 47 genomes from the environment, 4 genomes from plants, and the source of 7 genomes is unknown. To facilitate the analysis of the results, we have renumbered the genomes with excessively long names in the public database. The detailed information of the genomes is listed in Supplementary Table S1. Genomic DNA of *S. algae* strains was extracted using Bacterial Genome DNA Extraction Kit (DP302, Tiangen, China) according to the kit instructions. The whole genome of *S. algae* strains was sequenced using Illumina NovaSeq PE150 platform at the Beijing Novogene Bioinformatics Technology Co., Ltd. The initial sequencing data, referred to as raw data, contained a portion of low-quality data. To ensure the precision and dependability of subsequent data analysis, the raw data must be filtered to obtain valid data, known as clean data. Then, the clean data were assembled using SOAP *de novo* software (Honaas et al., 2016). A geographic distribution map of 206 genomes in this study was generated using the website.^2^ Based on existing literature, we also drew a map of the global geographic distribution of *S. algae* isolates.

### Average nucleotide identity analysis

We utilized the FastANI method to perform pairwise comparisons across all genomic sequences, resulting in the average nucleotide identity (ANI) values between any two genomes (Jain et al., 2018). The ANI values of 95% are accepted for species delineation.

### Pangenome and core genome analysis

The CD-HIT software was used for clustering analysis with a threshold of 50% pairwise identity and 0.7 length difference cutoff in amino acids to identify core genes, dispensable genes, and strain-specific genes (Huang et al., 2010). The resulting matrix was imported into R. Then, R Package “Pheatmap” was used to draw the pan-genome heatmap. The dilution curves were generated by randomly sampling combinations ranging from 1 to all available samples, followed by counting the number of core and pan-genome genes for each combination. Subsequently, boxplots for core and pan-genome in each combination were created, and finally, the dilution curves were plotted using the R package “boxplot.”

### Phylogenetic analysis

The amino acid sequences of the core genes were aligned using the MUSCLE software with a maximum number of iterations of 16. Alignments were trimmed from both ends. Phylogenetic tree based core genes sequence was reconstructed using the neighbor-joining methods by TreeBeST software (Vilella et al., 2009). Branch robustness was assessed by bootstrap analysis with 1,000 resamplings, and *p*-distance model was used to calculate evolutionary distances.

### Virulence, antibiotic resistance and plasmids genes and integrons analysis

Using the BLASTP alignment algorithm from the DIOMB software, the amino acid sequences of the genomes in this study were compared against the Virulence Factors of Bacterial Pathogens Database (VFDB) (Liu et al., 2019) and Comprehensive Antibiotic Research Database (CARD) (Alcock et al., 2023). The selection parameters of virulence analysis were set to an *E*-value less than 1 × 10^−5^, identity greater than or equal to 40%, and coverage greater than or equal to 40%. The top-scoring results were used as the final annotation outcomes of virulence-related genes. The selection parameters of antibiotic resistance analysis were set to an *E*-value less than 1 × 10^−5^, identity greater than or equal to 70%, and coverage greater than or equal to 40%. The top-scoring results were used as the final annotation outcomes of antimicrobial resistance genes. Transposons in each genome were predicted using the TransposonPSI software. Custom scripts were then used to compare the positions of the transposons with those of the resistance genes. If a transposon was found within 10k bp upstream or downstream of a resistance gene, both the resistance gene and the transposon were marked for their positional association. Using the BLASTn software to compare the assembled sequences with the sequences in the plsDB database (Schmartz et al., 2022), results that meet the criteria of an *E*-value less than 1 × 10^−5^ and a score greater than 10k, with both similarity and alignment coverage greater than 40%, are retained and considered as plasmids. Integrons identification was conducted using Integron Finder (version 2.0.5), with the distance threshold of 4,000 bp, and the *E*-value filtering threshold of 1 (Néron et al., 2022).

### Phenotypic antimicrobial susceptibility test

The susceptibility testing was performed according to the 2024 edition of the Clinical and Laboratory Standards Institute (CLSI-M100) guidelines for other non-Enterobacteriaceae, using the MIC method. Sixty-two *S. algae* strains from this study were tested for susceptibility to 17 types of antibiotics. Pure cultures grown to the logarithmic phase were picked from 3 to 5 single colonies and mixed in 0.45% NaCl solution to prepare a bacterial suspension with a 0.5 McFarland turbidity standard, resulting in a 3 mL suspension. Then, 145 μL of the above bacterial suspension was diluted in 3 mL of 0.45% NaCl solution to the standard concentration. The AST-335 antimicrobial susceptibility card was inserted, and the card rack was loaded onto the VITEK 2 Compact Automated Microbial Identification and Antimicrobial Susceptibility Testing System to monitor bacterial growth in each well. After the incubation period, the antibiotic susceptibility test results for each antimicrobial agent on the susceptibility card were obtained from the automated system VITEK 2 device. *Pseudomonas aeruginosa* ATCC 27853 was used as the quality control strain.

## Results

### General genomic characteristics

**Figure 1:**
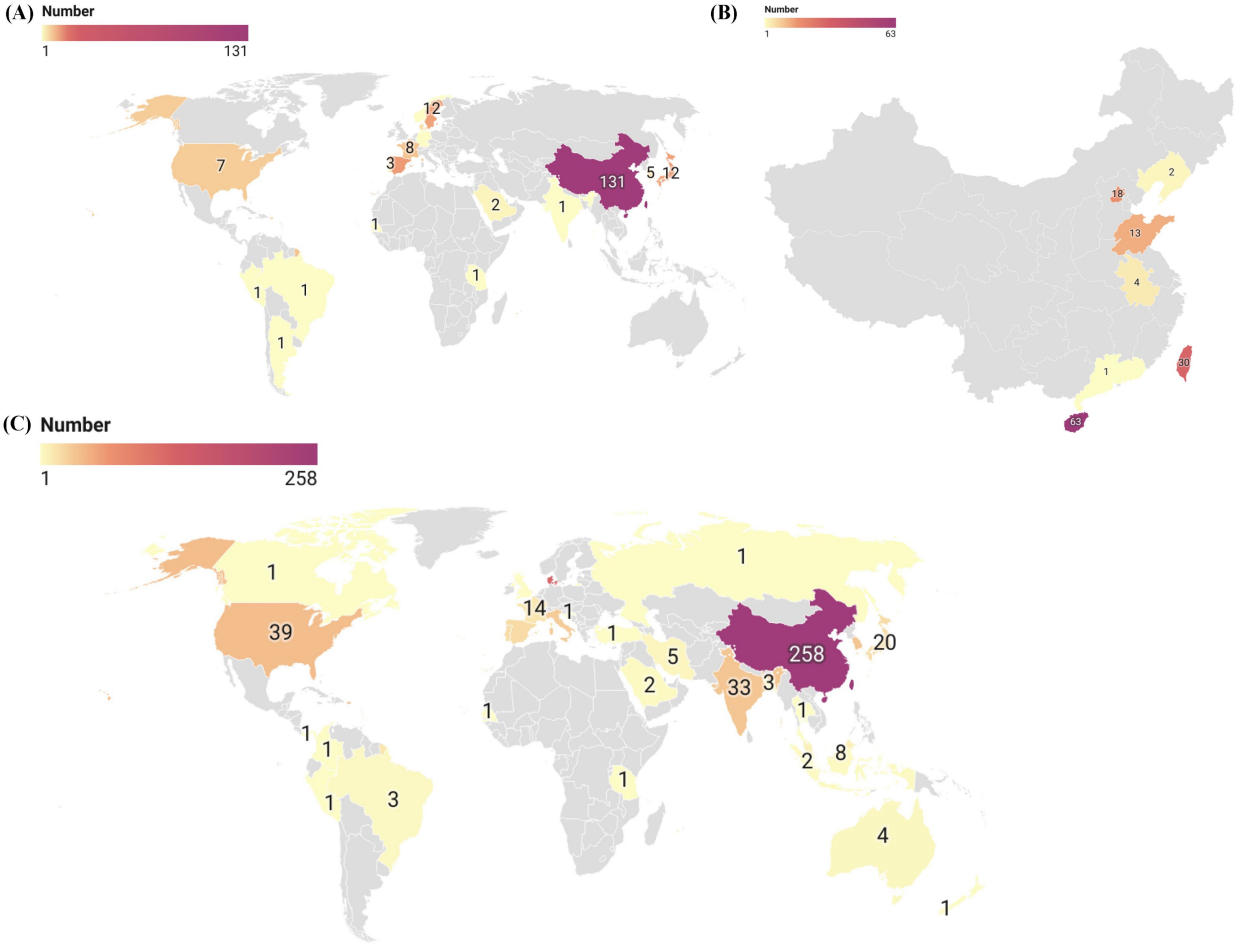
The global geographic distribution map of *S. algae* genomes and isolates. **(A)** In this study, a global geographic distribution map of 206 *S. algae* genomes. **(B)** A geographic distribution map of 131 *S. algae* genomes originating from China. **(C)** A global geographic distribution map of *S. algae* isolates based on existing literature.

The geographic distribution of the 206 *S. algae* genomes from various countries is shown in Figure 1A, while the distribution of the genomes originating from China across different provinces is depicted in Figure 1B. Additionally, we conducted a full-text search using the search terms “*Shewanella algae*” or “*Shewanella haliotis*” or “*Shewanella upenei*” which are confirmed to be *S. algae*, and a total of 295 articles were identified after the database search. We extracted the information on the sources of *S. algae* strains and created a geographic distribution map of countries from which *S. algae* strains have been isolated in Figure 1C. It was found that there are 626 reported *S. algae* strains isolated worldwide, distributed across 30 countries, with the highest number of isolates originating from China, totaling 258 strains. Most genomes and isolates were from coastal countries and cities. The genomic characteristics of the 206 genomes of *S. algae* demonstrated variability in both genome size, ranging from 4.60 kb in the CLS3 genome to 5.20 kb in the KCNaR1 genome, and G + C content, which varied from 52.49 to 53.18%. Detailed information for these genomes can be found in Supplementary Table S2.

**Figure 2:**
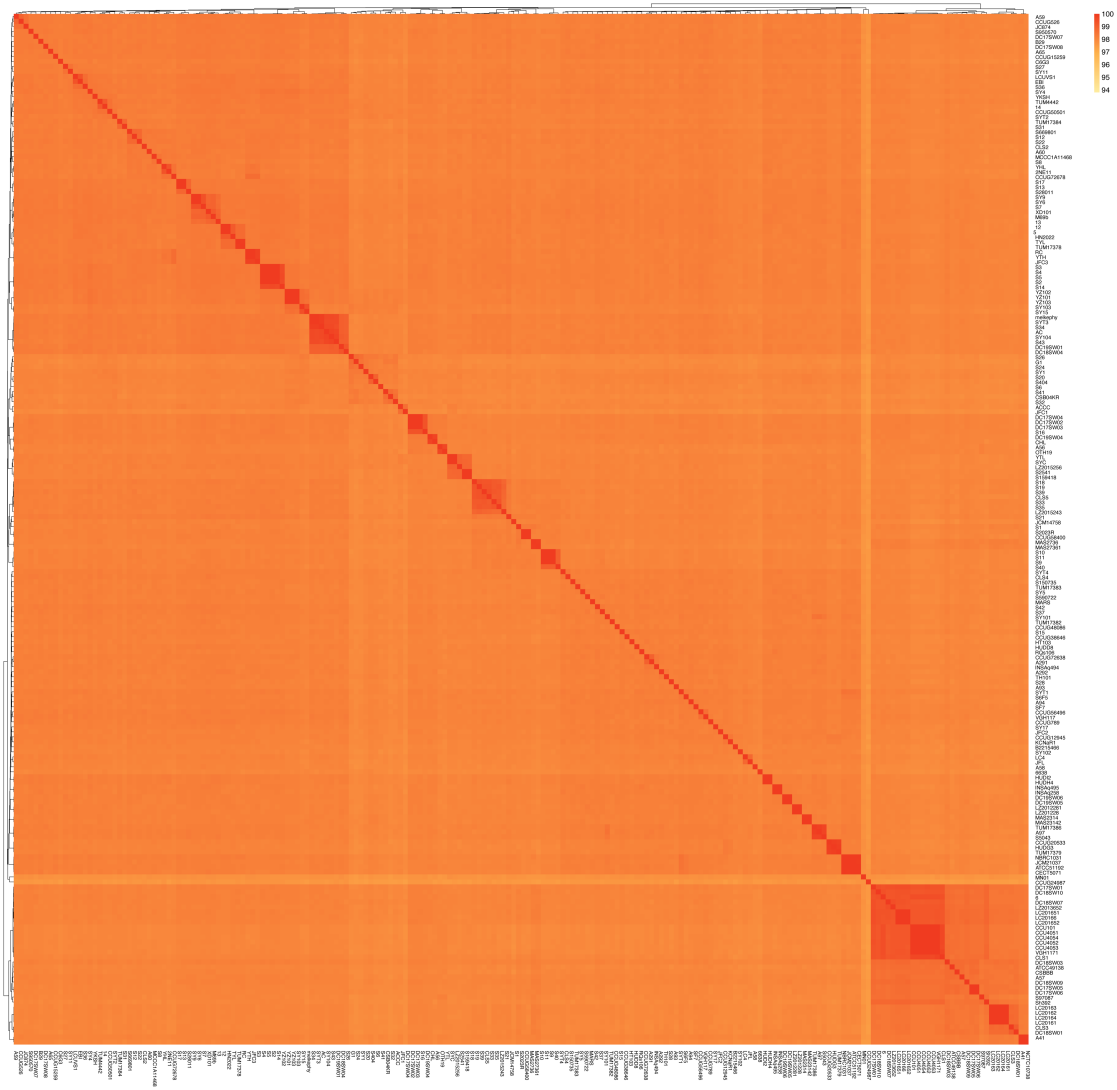
Average nucleotide identity (ANI) values for the genomic sequences of the 206 *S. algae*.

A dendrogram illustrating the genetic relationships among these genomes was constructed using a matrix based on average nucleotide identity (ANI) values, as depicted in Figure 2. The ANI values ranged from 97.34 to 100%, indicating that these genomes all belong to the *S. algae*. Particularly, higher ANI values (99.99–100%) were observed within these genomes/strains (5/12, S2/S3/S4/S5, S9/S10/S11, S13/S17, SY6/SY9, YZ101/YZ102/YZ103, VGH1171/CCU101/CCU4051/CCU4052/CCU4053/CCU4054, NCTC10738/A41, SYC/YTL, YTH/RC, HUDH4/HUDI2, S159418/S2541, DC19SW06/DC19SW05, LC201651/LC201652/LC20166, DC17SW02/DC17SW03/DC17SW04, LC20161/LC20162/LC20163/LC20164). Based on a definition threshold for strain at 99.99% ANI, they are likely to be the same strain (Rodriguez et al., 2024). Strains 6,638, 6, and S1–S6 were derived from five clinical patients in this study. Specifically, strains S2–S5 were isolated from the same individual, and they are a clone. Three of the patients had a history of exposure to marine environments. Comprehensive clinical diagnostic and treatment information is provided in Table 1.

**Table 1:** Characteristics of patients with *S. algae* infection.

Case	Sex	Age	Specimen sources	Isolated strain	Clinical diagnosis	Treatment	Outcome	Marine exposure
1	Male	27	Wound secretions	6,638	Right index finger distal amputation injury, closed fracture of the proximal phalanx of the right middle finger, multiple abrasions and lacerations on the right hand	Replantation of the right index finger, closed reduction and external fixation of the proximal phalanx of the right middle finger, debridement and suturing of the right hand, postoperative infection prophylaxis and symptomatic supportive treatment	Necrosis of the replanted right index finger, wound healing improves	Suffered entanglement and injury from machinery ropes during fishing operations in the ocean
2	Male	57	Wound secretions	6	Right-sided osteomyelitis of the foot, infected diabetic foot on the right side, type 2 diabetes mellitus	Right foot wound debridement, vacuum-assisted closure (VAC) therapy, flap repair surgery, anti-infective treatment, blood sugar regulation and symptomatic supportive treatment	Improvement	Beach walk
3	Female	66	Sputum	S1	Acute exacerbation of chronic obstructive pulmonary disease, bronchiectasis with infection, nodule in the right upper lobe of the lung, right pulmonary space occupying	Anti-infective, expectorant, and anti-asthmatic therapy	Improvement	Unknown
4	Male	71	Sputum	S2, S3, S4, S5	Acute respiratory failure, severe pneumonia, chronic obstructive pulmonary disease, acute large area cerebral infarction, multiple organ dysfunction syndrome, septic shock	Cerebral angiography, intracranial angioplasty and stenting, postoperative mechanical ventilation, anticoagulation, antiplatelet therapy, prophylaxis for cerebral edema, anti-infective treatment, and symptomatic supportive care	The patient’s condition is critical, yet the family insists on taking the patient out of the hospital	Unknown
5	Male	27	Wound secretions	S6	Open fracture of the right lower leg with associated muscle and tendon ruptures, soft tissue loss in the right lower leg	Debridement and exploratory repair surgery of the right lower leg, postoperative anti-infection, analgesia, and other symptomatic supportive treatments	Improvement	Injured by a motorboat collision during marine recreational activities

### Pangenome and core genome analysis

**Figure 3:**
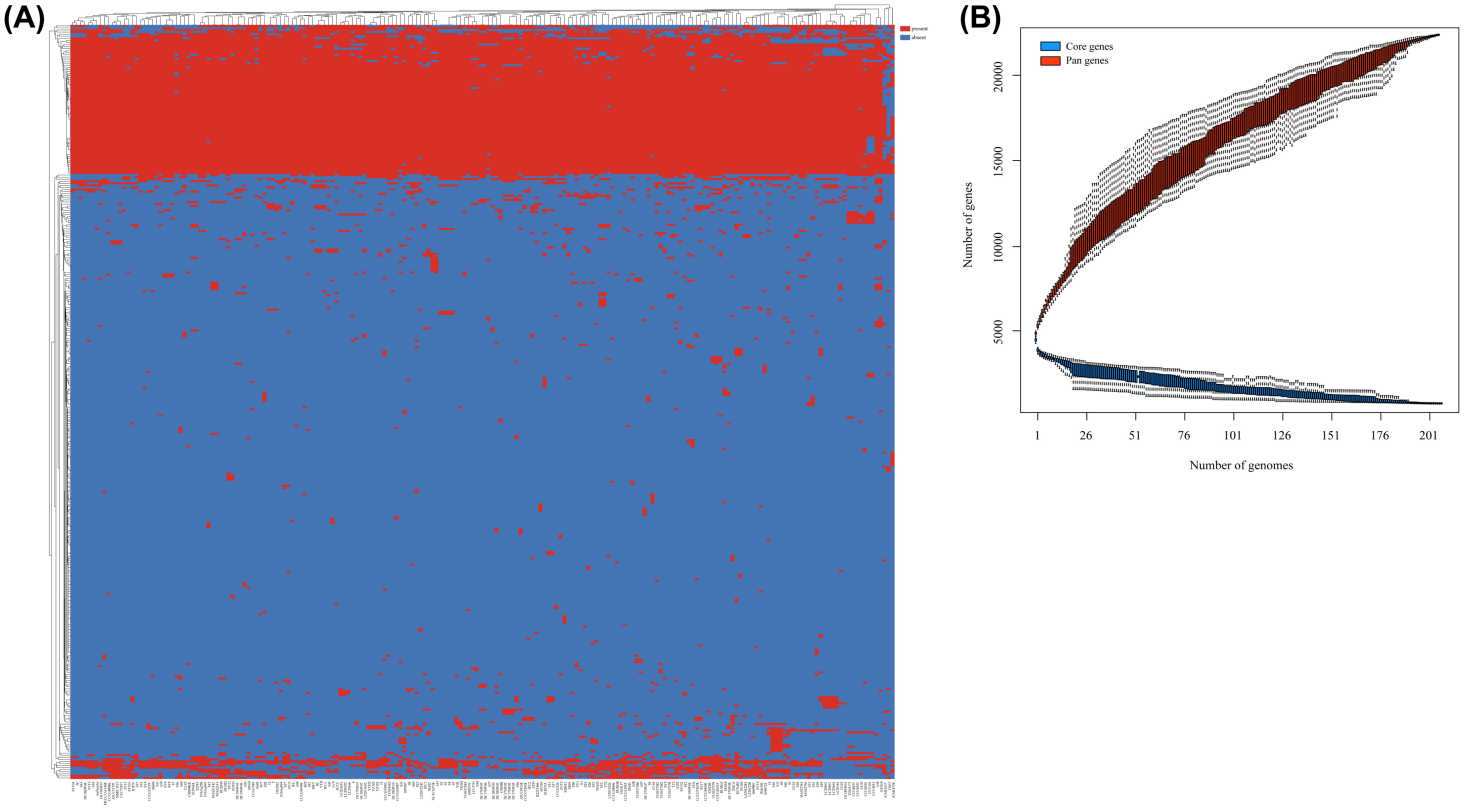
Pan-genome analysis of 206 *S. algae*. **(A)** The heatmap based on pan-genome sequences. The top represented genome names, and the left side represented pan-genes clusters. Red indicated the presence of genes, while blue indicated the absence of genes. **(B)** The core/pan-genome dilution curves. Red represented core genes, while blue represented pan genes.

In this study, we identified a total of 21,909 genes across 206 *S. algae* genomes, constituting their collective pan-genome (Figure 3A). The number of CDSs varied, with the lowest count of 4,193 in CLS3 genome and the highest of 5,917 in C6G3 genome. A total of 1,563 genes were common to every genomes, forming the core genome. The core genome’s COG classification highlights genes that are crucial for basic biological functions, such as translation, posttranslational modification, and energy generation. The core genome spans a length of 1,893,053 bp, with a total of 910,124 single nucleotide polymorphisms (SNPs) identified. Using the complete genome of 2NE11 as a reference, the SNP count varied significantly, from 27,492 in YHL genome to 97,576 in CCUG24987 genome, with an average of 77,218 SNPs per genomes. Additionally, the pan-genome’s dilution curve revealed a continuous increase in novel gene families with the inclusion of more genomes, contrasting with the core genome’s gene count, which tended to stabilize over time (Figure 3B). Among the 206 *S. algae* genomes, the count of unique genes varied significantly, with numbers ranging from 0 to 2,371. The clinical genomes exhibited an average of 42 unique genes, whereas animal and environmental genomes had an average of 124 and 71 unique genes, respectively (Supplementary Figure S1).

### Phylogenetic analysis

**Figure 4:**
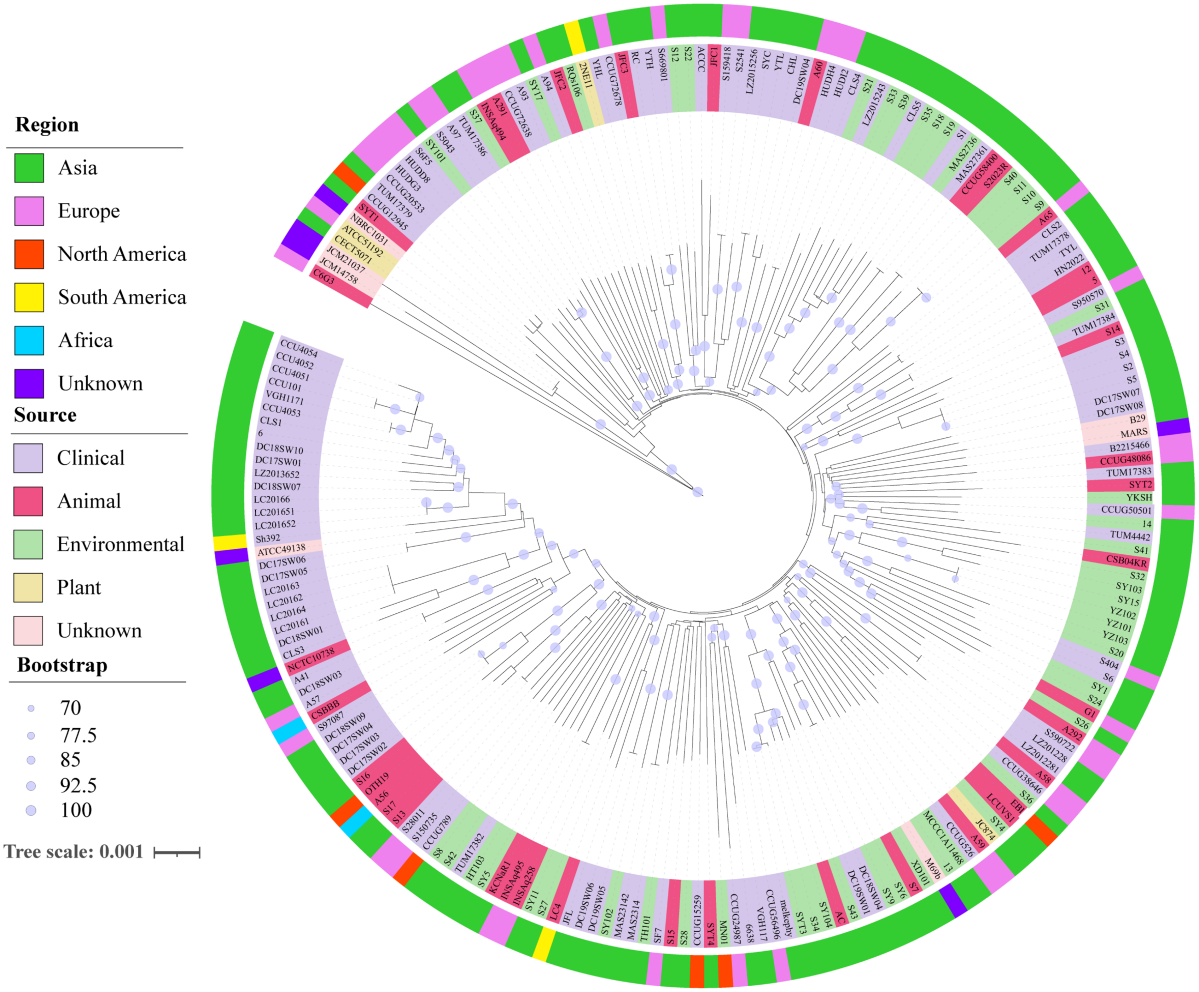
Neighbor-joining phylogenomic tree of 206 *S. algae* based on core genes. The outer ring with different colors represented different regional genomes, while the inner ring with different colors represented different sources of genomes. The robustness of the tree structures was assessed using 1,000 resamples. The scale corresponds to a nucleotide substitution rate of 0.001 mutations per site.

A neighbor-joining phylogenetic tree was constructed using robust core genome sequences to explore the evolutionary relationships among 206 genomes of *S. algae* (Figure 4). On the phylogenetic tree, genomes from same geographical origins did not show distinct clustering, and genomes from different geographical origins also have close phylogenetic relationships, suggesting that *S. algae* has a wide prevalence across regions. Some genomes from different sources were found to have close phylogenetic relationships. For example, genomes from the environment and animals (KCNaR1/SY5, AC/SY104, SY9/SY6/S7, LCUVS1/EBI/S36, CSB04KR/S32/S41, YKSH/SYT2), genomes from the environment and clinical patients (TUM17382/HT103, DC19SW05/DC19SW06/SY102, MN01/CCUG24987/6,638, melkephy/SYT3/S34, S6/SY1/S24/S26, S20/S404, CCUG50501/TUM4442/14, TUM17384/S31/S950570, MAS27361/MAS2736/S1, S35/CLS5, SY17/A93, TUM17386/A97/S5043/SY101), genomes from animals and clinical patients (NCTC10738/A41, DC18SW03/A57/CSBBB, CCUG38646/A58, LZ2012281/LZ201228/S590722/A292, B2215466/CCUG48086/TUM17383, S2/S3/S4/S5/S14, HN2022/5/12, CLS2/A65, HUDI2/HUDH4/A60, JFC1/ACCC, CCUG72638/A291/INSAq494), genomes from the environment, animals, and clinical patients (SF7/TH101/S15, CCUG15259/S28/SYT4, RQs106/JFC2/A94), and genomes from the environment, plants, animals, and clinical patients (CCUG526/A59/JC874/SY4). The pairwise SNP distance distribution (Supplementary Figure S2) indicated that genomes within Clade 1 exhibited a more closely related evolutionary connection compared to those in Clades 1 and 3.

### Prediction of virulence-associated genes and plasmids

**Figure 5:**
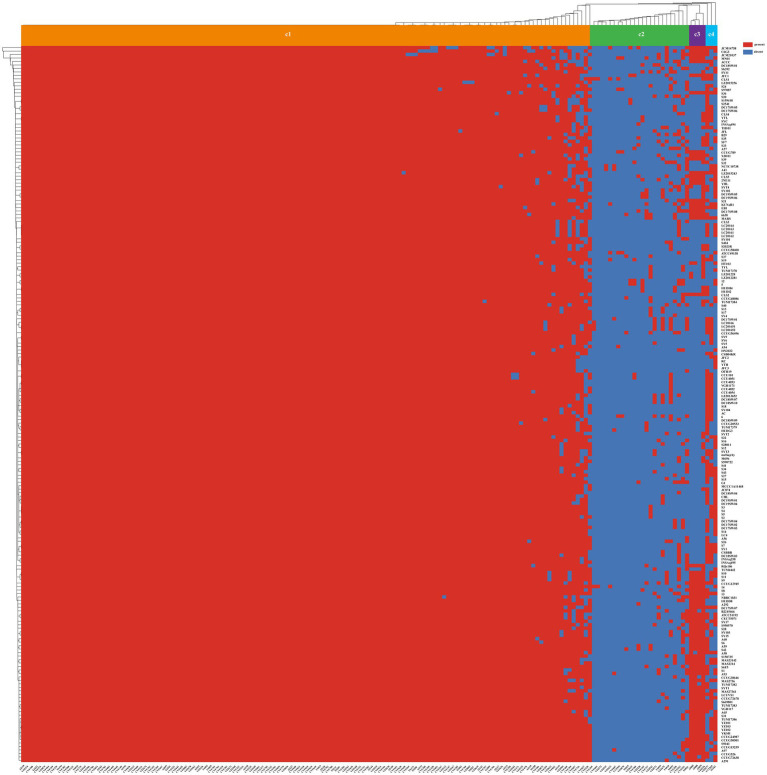
The heatmap of the virulence factors in 206 *S. algae*. Above the heatmap, the virulence factors clusters were labeled from Cluster 1 to Cluster 5. Virulence factors ID number were shown at the bottom of the heatmap, and the renamed genomes were shown to the right of the heatmap. The presence of virulence factors was denoted by red, while its absence was indicated by blue.

A diverse array of putative virulence-associated genes was identified, spanning 14 category and 16 subcategory with 171 distinct virulence factors (Supplementary Table S3). The virulence-associated genes can be classified into four clusters (Figure 5). The genomic analysis revealed the presence of 133 to 151 known or putative virulence factors across the genomes. Most genomes are equipped with potential virulence genes associated with fimbrial adhesin, antimicrobial activity/competitive advantage, biofilm formation, effector delivery system, antiphagocytosis, complement evasion/serum resistance, inflammatory signaling pathway, motility, iron uptake, post-translational modification, regulation, exoenzyme, exotoxin and stress survival. But they rarely include genes from Cluster 2 to Cluster 4 that are responsible for the Lvh T4SS, Trw T4SS, Pyoverdine, Pyochelin, coxH2/rimL T4SS, CdpA, AAI/SCI-II T6SS and MSHA type IV pili virulence factors. By comparison with the VFDB database, *S. algae* has been found to possess complete corresponding putative virulence genes in the following virulence factors, including the Carbon storage regulator A (*csrA*), Macrophage infectivity potentiator (*mip*), Autoinducer-2 (*luxS*), Cysteine acquisition (*ggt*), Listeria adhesion protein (*lap*), Glyceraldehyde-3-phosphate dehydrogenase (*plr*, *gapA*), Streptococcal enolase (*eno*), FAS-II (*kasB*), Isocitrate lyase (*icl*), Leucine synthesis (*leuD*), Glutamine synthesis (*glnA1*), AhpC (*ahpC*), Hsp60 (*htpB*), RelA (*relA*), Zmp1 (*zmp1*), Copper exporter (*ctpV*), Nucleoside diphosphate kinase (*ndk*), Catalase (*katA*), PbpG (*pbpG*), Hemolysin HlyA (*hlyA*), Hemolysin III (AHML_18530), EF-Tu (*tufA*), AcrAB (*acrA*, *acrB*), ClpE (*clpE*), ClpP (*clpP*), SodCI (*sodCI*), RpoS (*rpoS*), Fur (*fur*), GspA (*gspA*), SodB (*sodB*), GbpA (*gbpA*), CdpA (*cdpA*), Haemophilus iron transport locus (*hitA*, *hitB*, *hitC*), Pyrimidine biosynthesis (*carA*, *carB*, *pyrB*), KatAB (*katA*, *katB*). Plasmids were detected in 33 genomes, with 19 genomes from clinical samples, 6 from animal samples, 5 from environmental sources, 2 from plants, and the remaining 1 genome with an unknown source. Three distinct plasmid replicon types were identified. The information of antimicrobial resistance genes and the roles of the proteins encoded by genes associated with virulence were detailed (Supplementary Table S4).

We identified 53 complete integrons, 216 CALIN, and 83 In0 in 181 of the 206 *S. algae* genomes, resulting in a (CALIN+In0)/complete ratio of 5.64 (Supplementary Table S5). As our dataset predominantly consists of draft genomes, it was anticipated that some complete integrons might be split across multiple contigs, resulting in a higher percentage of CALIN and In0.

### Potential antimicrobial resistance genes of *Shewanella algae*

In the *S. algae* genomes, we identified genes conferring resistance to 10 different antibiotic classes (Supplementary Table S6). Resistance genes targeted β-lactams (such as *bla_CMY-2_, bla_NDM-1_, bla_OXA-4_, bla_OXA-10_, bla_VEB-1_, bla_TEM-208_, bla_TEM-102_, bla_TEM-2_, bla_TEM-210_, bla_CARB-3_, bla_CTX-M-103_*), aminoglycosides (*aac(3)-Ib, aac(3)-IIb, acrD, ant(3″)-IIa, aph(6)-Id, armA, aadA, aac(3)-IIa, aac(6′)-Ib4, ant(2″)-Ia, aadA3, aadA2, aadA16, aadA7, aph(3′)-Ia, aph(3″)-Ib, aph(6)-Id*), quinolones (*qnrD1, qnrA1, qnrA2, qnrA3, qnrA6*), phenicols (*cmlA5, catI, catB3, floR*), macrolides (*mphA*), sulfonamides (*dfrA1, dfrA27, dfrA36, rsmA, sul1, sul2*), tetracyclines (*tet(K), tet(A), tet(D), tet(59)*), and multidrug resistance efflux pumps (*crp*). The main resistance gene targeted by polypeptides is *ugd*; for sulfonamides, it is *rsmA*; and for aminoglycosides, the main resistance genes are *aac(3)-IIb*, *acrD*, and *emrE*. The predominant genotype of *qnr* and *bla_OXA_* genes are *qnrA3* and *bla_OXA-405_*, *respectively*. An analysis of the genetic environment of the identified resistance genes has been conducted. Sixty-three genomes were found to have mobile genetic elements (MGEs) within the 10 kb sequences upstream and downstream of the resistance genes (Supplementary Table S7).

### Antibiotic resistance screening of *Shewanella algae* strains

The results of the antimicrobial susceptibility testing for the newly isolated 62 strains of *S. algae* in this study can be found in Supplementary Table S8. All strains were sensitive to amikacin, minocycline and tigecycline, but exhibited a high rate of resistance to imipenem and colistin (Table 2). Strain isolates from clinical, animal, and their living water pool sources all showed varying degrees of drug resistance, with the strains 06638, 13, 14, S7 and S12 exhibiting multidrug resistance (insensitive to three or more types of antibiotics). Specifically, strains S7 and S12 isolated from Hawksbill and its living environment, respectively, and possess a unique resistance profile.

**Table 2:** The results of antimicrobial resistance rate of 62 *S. algae* strains.

Antibiotics	Rate of resistance (%)	Rate of intermediate resistance (%)	Rate of susceptibility (%)
TCC	3.23	0	96.77
TZP	3.23	1.61	95.16
CAZ	3.23	0	96.77
CPS	3.23	0	96.77
FEP	0	4.84	95.16
ATM	1.61	0	98.39
IPM	48.39	1.61	50
MEM	3.23	0	96.77
AMK	0	0	100
TM	3.23	3.23	93.55
CIP	6.45	1.61	91.94
LEV	0	1.61	98.39
DO	1.61	0	98.39
MNO	0	0	100
TGC	0	0	100
CS	43.55	0	56.45
SXT	8.06	0	91.94

TCC, ticarcillin/clavulanic acid; TZP, piperacillin/tazobactam; CAZ, ceftazidime; CPS, cefoperazone/sulbactam; FEP, cefepime; ATM, aztreonam; IPM, imipenem; MEM, meropenem; AMK, amikacin; TM, tobramycin; CIP, ciprofloxacin; LEV, levofloxacin; DO, doxycycline; MNO, minocycline; TGC, tigecycline; CS, colistin; SXT, trimethoprim/sulfamethoxazole. *Pseudomonas aeruginosa* ATCC 27853 was used as a control.

## Discussion

This study is the first to find *S. algae* infections in clinical patients and animals in Hainan Province, as well as isolating *S. algae* from the marine environment. Among them, three patients had a history of marine exposure, which is consistent with previous studies (Ng et al., 2022). In two other patients, it was the first time that *S. algae* were isolated from sputum samples, but the history of their marine exposure was unclear. Temperature plays a crucial role in the pathogenicity of *S. algae* (Tseng et al., 2018). We isolated *S. algae* from the marine environment across different seasons, with no significant difference in the isolation rates, which may be related to the warm climate and minimal temperature variation throughout the year in Hainan Province (Liu et al., 2013; Torri et al., 2018; Tseng et al., 2018).

A recent study has recently proposed a definition threshold for strain at 99.99% ANI (Rodriguez et al., 2024). This indicates that among the clinical strains isolated in this study, all except for S2–S5 (which were isolated from the same patient and are clonally identical) are different strains. In other words, *S. algae* strains from different patients are not clonally identical. Similarly, *S. algae* strains from different parts of the same dolphin (strains 5 and 12) and porcupine fish (strains S13 and S17) are the same. We found that some genomes isolated from patients at different times also have an ANI value exceeding 99.99%. This high degree of genetic similarity suggests that *S. algae* can cause recurrent infections in patients (Vogel et al., 2000). Genomes MAS2314/MAS23142 (with the original strain numbers both being 08MAS2314), genomes ATCC51192/CECT5071 (Tellgren-Roth et al., 2021) are the same strain, yet their ANI values are 99.983 and 99.984% respectively, which is below the strain threshold.

The phylogenetic analysis results of this study are consistent with previous research, with genomes having high ANI similarity located on the same evolutionary branch (Fang et al., 2019; Huang et al., 2022). Most genomes from the marine environment have a close phylogenetic relationship with those isolated from patients and marine animals. In addition, we found that genomes from clinical patients in Japan, Sweden, Spain, Denmark, and Beijing and Taiwan in China were closely related to some genomes from marine environmental samples in this study, further confirming that marine environmental exposure is a risk factor for *S. algae* infection (Yu et al., 2022). Therefore, it is important for people to take precautions when exposed to the marine environment to avoid trauma that can cause *S. algae* infection.

In addition, *S. algae* has been isolated from a variety of animals. Some of which are commonly eaten seafood. Consuming raw fish is considered to be a route of transmission for this pathogen (Holt et al., 2005; Ng et al., 2022). For example, strains S2–S5 patient isolate in this study was related to the local strain S14 isolated from the spiny dolphin, suggesting the possibility that humans may be infected with the bacterium through food marine animals. A phylogenetic tree also showed a cluster within strains S2–S5 from the patient in this study and strain S14 from local *Diodon holacathus*. This suggests the possibility that the patients may have been infected through the consumption of seafood. From the strain isolation source information, it is also confirmed that *S. algae* is widespread in the marine environment of temperate tropical countries, and can cause infection in humans, marine animals, and plants.

Pan-genome analysis delved into the genetic reservoir, unique genes, and functional insights driving bacterial diversification. The accessory genes outnumbered core genes by a factor of 7.7, highlighting the substantial genomic diversity of *S. algae* (Huang et al., 2022). The COG function annotation revealed that the unique genes in *S. algae* were predominantly associated with signal transduction mechanisms, suggesting that accessory genes, particularly those unique genes acquired from various environments, might play a significant role in enhancing the survival capabilities of *S. algae* (Supplementary Figure S3).

Currently, the toxicity of *S. algae* has been confirmed *in vitro* assays (Beleneva et al., 2009), but the pathogenic mechanism remains unclear. Some studies have found that the virulence genes of *S. algae* are related to metalloproteases, flagella, capsule polysaccharide biosynthesis, T2SS, T6SS, heme biosynthesis, and outer membrane heme receptors, which is consistent with our research (Heidelberg et al., 2002; Huang et al., 2022). Quorum sensing is a form of cell-to-cell communication among bacteria that can activate the transcription of specific genes, thereby regulating the physiological characteristics of microbes (Whiteley et al., 2017). It typically includes the AHLs type, LuxS/AI-2 type, and AI-3 type. All of our strains contain genes involved in the synthesis of AI-2 (*LuxS*), but we have not found genes encoding AI-2 receptors (*LuxP* or *LsrB*), indicating that these strains cannot perceive AI-2. The LuxS/AI-2 QS system may participate in competition, cooperation, or communication within microbial communities by synthesizing and secreting the AI-2 signaling molecule (Li et al., 2020). Bacterial secretion systems are membrane-anchored nanomachines that enable bacteria to transport various effector proteins from the cell into the surrounding niche or directly into the cytoplasm of eukaryotic/prokaryotic cells, playing a key role in bacterial pathogenic mechanisms (Green and Mecsas, 2016). Contrary to previous research (Huang et al., 2022), we have found that most of our strains (including clinical strains) possess a cluster of 17 homologous genes that are characteristic of the VAS T6SS family (including *vgrG-1*, *vgrG-2*, *hcp-2*, *vipA*, *vipB*, *hsiF*, *vasA*, *vasB*, *vasC*, *vasD*, *vasE*, *vasF*, *clpB*/*vasG*, *vasH*, *vasI*, *vasJ*, *icmF*/*vasK*), indicating the presence of the VAS T6SS in our bacterial strains. VAS T6SS exhibits both antibacterial and antieukaryotic activities (Ishikawa et al., 2012; Dong et al., 2013).

Consistent with other research findings, all genomes contained *RpoS*, stress response regulators *sodCI*, *katA* and *katB*, *hlyA*, and *hlyIII*. *RpoS* is an important global regulatory factor involved in the formation of biofilms in *Pseudomonas fluorescens* (Liu et al., 2018). The *sodCI* gene encodes superoxide dismutase, an enzyme capable of breaking down free radicals produced by host cells, thereby neutralizing the bacteria and enhancing their resistance (Krishnakumar et al., 2007). *katA* and *katB* genes encode the two catalase-peroxidases and are essential for maintaining a critically low concentration of H_2_O_2_ by balancing the levels of H_2_O_2_ between the periplasmic space and the cytoplasm, which is important for intracellular survival and transmission (Bandyopadhyay et al., 2003). The *hlyIII* encodes an ABC transporter that secretes *hlyA*, and *hlyA* can encode the assembly of the RTX toxin protein α-hemolysin, which alters the permeability of the cell membrane, causing lysis of cells in the patient or animal host (Wang et al., 2020). We also identified virulence factors associated with bile tolerance (Begley et al., 2005; Okoli et al., 2007), such as the presence of the *PhoPQ* regulatory in all genomes, the *htpB* gene in most genomes, and the *wecA* gene in 92 genomes, which are consistent with previous studies. Additionally, studies have also identified genomic island mobile elements that contribute to *S. algae*’s acquisition of virulence factors (Huang et al., 2022).

To date, there are no clear diagnostic and treatment guidelines for *S. algae* infections, and its resistance mechanisms are not fully understood. *S. algae* is considered a repository and/or vectors for resistance genes, exhibiting resistance to first-and second-generation cephalosporins, penicillins, colistin, and carbapenems (Cimmino et al., 2016). This study found that three strains derived from animals and the environment are resistant to third-and fourth-generation cephalosporins and penicillin, and 27 strains are resistant to colistin. Due to the presence of class D β-lactamases, *S. algae* has developed new resistance to imipenem and piperacillin/tazobactam (Cimmino et al., 2016). Approximately half of the strains isolated in this study were resistant to imipenem, and three were resistant to piperacillin/tazobactam. *S. algae* is considered to be the origin of the plasmid-mediated quinolone resistance gene *qnrA* (Nordmann and Poirel, 2005). This study found resistance to fluoroquinolones in five strains, most of which were clinical and animal strains. Almost all strains in this study carried resistance genes to quinolones, sulfonamides, *β*-lactams, aminoglycosides, and polypeptides, as well as efflux pump genes. However, the resistance phenotype and resistance genes of the strains did not completely match, which may be related to the incompleteness of the strain genomes. Other studies have also shown that there is no complete consistency between resistance phenotypes and resistance genes (Ho et al., 2013; Pornsukarom et al., 2018). Additionally, we identified 352 integrons (complete and/or incomplete) across 181 genomes, of which 53 were complete. Integrons play a significant role in the emergence and dissemination of antimicrobial resistance genes in *S. algae* (Ayala Nuñez et al., 2022; Chia-Wei et al., 2022).

There is an increasing number of reports on infections caused by *S. algae* in both humans and animals, highlighting the importance of this pathogen in zoonotic diseases. Further research is needed to explore the genetic evolutionary relationships among strains isolated from different clinical manifestations, hosts, and regions. To fully understand the pathogenic mechanisms of this zoonotic pathogen, more genomic information of *S. algae* is required, especially from strains isolated from rare host species. Additionally, investigating the spread of *S. algae* between countries and regions is crucial for a better understanding of the global epidemiology of *S. algae*.

## Conclusion

This study is novel in that it is the first to report the presence of *S. algae* infections in patients and animals in Hainan. Our findings clarify the genomic characteristics and population structure of *S. algae* in Hainan Province. The results offer insights into the genetic basis of pathogenicity in *S. algae* and enhance our understanding of its global phylogeography. *S. algae* should be considered an emerging opportunistic pathogen linked to environmental conditions in the coastal area. Therefore, it is an urgent call for surveillance and control activities against *S. algae* infections.

## Data Availability

The datasets presented in this study can be found in online repositories. The names of the repository/repositories and accession number(s) can be found in the article/Supplementary material.

## Footnotes

^1^http://www.bacterio.net/shewanella.html

^2^https://app.datawrapper.de
