# Supplementary material for: Genome characterization of Shewanella algae in Hainan Province, China
Source: Front Microbiol. 2024 Oct 2;15:1474871. doi: 10.3389/fmicb.2024.1474871 (PMC11480045; doi:10.3389/fmicb.2024.1474871)
Supplement: Supplementary file 1 [file Data_Sheet_1.pdf]

Supplementary files

Table S1. Detailed information of 206 *S. algae* strains

NO.	Strain	Renamed strain	Isolation source	Collection year	Collection Region	Host	Host disease	Assembly Accession
1	6638	—	Wound secretions from patient	2019	Sanya city, Hainan Province, China	Homo sapiens	Infection	JBDIMM000000000
2	6	—	Wound secretions from patient	2019	Sanya city, Hainan Province, China	Homo sapiens	Infection	JBDIMN000000000
3	5	—	Wound secretions	2018	Sanya city, Hainan Province, China	Dolphin	Infection	JBDIMO000000000
4	12	—	Wound secretions	2018	Sanya city, Hainan Province, China	Dolphin	Infection	JBDIMP000000000
5	13	—	Wound secretions	2019	Sanya city, Hainan Province, China	Dolphin	Infection	JBDIMQ000000000
6	14	—	Wound secretions	2019	Sanya city, Hainan Province, China	Dolphin	Infection	JBDIMR000000000

7	S1	—	Sputum from patient	2021	Sanya city, Hainan Province, China	Homo sapiens	Infection	JBDIMS000000000
8	S2	—	Sputum from patient	2021	Sanya city, Hainan Province, China	Homo sapiens	Infection	JBDIMT000000000
9	S3	—	Sputum from patient	2021	Sanya city, Hainan Province, China	Homo sapiens	Infection	JBDIMU000000000
10	S4	—	Sputum from patient	2021	Sanya city, Hainan Province, China	Homo sapiens	Infection	JBDIMV000000000
11	S5	—	Sputum from patient	2021	Sanya city, Hainan Province, China	Homo sapiens	Infection	JBDIMW000000000
12	S6	—	Wound secretions from patient	2022	Sanya city, Hainan Province, China	Homo sapiens	Infection	JBDIMX000000000
13	S7	—	Foreign body in the lung	2021	Sanya city, Hainan Province, China	Hawksbill	Infection	JBDIMY000000000
14	S8	—	Artificial pools	2021	Sanya city, Hainan Province, China	—	—	JBDIMZ000000000
15	S9	—	Artificial pools	2021	Sanya city, Hainan Province, China	—	—	JBDINA000000000
16	S10	—	Artificial pools	2021	Sanya city, Hainan Province, China	—	—	JBDINB000000000
17	S11	—	Artificial pools	2021	Sanya city, Hainan Province, China	—	—	JBDINC000000000

18	S12	—	Artificial pools	2021	Sanya city, Hainan Province, China	—	—	JBDIND000000000
19	S13	—	Wound secretions	2020	Sanya city, Hainan Province, China	Diodon holacathus	Infection	JBDINE000000000
20	S14	—	Wound secretions	2020	Sanya city, Hainan Province, China	Diodon holacathus	Infection	JBDINF000000000
21	S15	—	Wound secretions	2020	Sanya city, Hainan Province, China	Diodon holacathus	Infection	JBDING000000000
22	S16	—	Wound secretions	2020	Sanya city, Hainan Province, China	Diodon holacathus	Infection	JBDINH000000000
23	S17	—	Wound secretions	2020	Sanya city, Hainan Province, China	Diodon holacathus	Infection	JBDINI000000000
24	S18	—	Seawater from Fengjia Bay	2021	Wenchang city, Hainan Province, China	—	—	JBDINJ000000000
25	S19	—	Seawater from Qishui Bay	2021	Wenchang city, Hainan Province, China	—	—	JBDINK000000000
26	S20	—	Seawater from Bamen Bay	2021	Wenchang city, Hainan Province, China	—	—	JBDINL000000000
27	S21	—	Seawater from Gaolong Bay	2021	Wenchang city, Hainan Province, China	—	—	JBDINM000000000

28	S22	—	Seawater from Hainan Bay	2021	Wenchang city, Hainan Province, China	—	—	JBDINN000000000
29	S24	—	Seawater from Gaolong Bay	2021	Wenchang city, Hainan Province, China	—	—	JBDINO000000000
30	S26	—	Seawater from Moon Bay	2021	Wenchang city, Hainan Province, China	—	—	JBDINP000000000
31	S27	—	Seawater from Bamen Bay	2021	Wenchang city, Hainan Province, China	—	—	JBDINQ000000000
32	S28	—	Seawater from Bamen Bay	2021	Wenchang city, Hainan Province, China	—	—	JBDINR000000000
33	S31	—	Seawater from Qishui Bay	2021	Wenchang city, Hainan Province, China	—	—	JBDINS000000000
34	S32	—	Seawater from Qishui Bay	2021	Wenchang city, Hainan Province, China	—	—	JBDINT000000000
35	S33	—	Seawater from Moon Bay	2021	Wenchang city, Hainan Province, China	—	—	JBDINU000000000

36	S34	—	Seawater from Fengjia Bay	2021	Wenchang city, Hainan Province, China	—	—	JBDINV000000000
37	S35	—	Seawater from Moon Bay	2021	Wenchang city, Hainan Province, China	—	—	JBDINW000000000
38	S36	—	Seawater from Hainan Bay	2021	Wenchang city, Hainan Province, China	—	—	JBDINX000000000
39	S37	—	Seawater from Bamen Bay	2021	Wenchang city, Hainan Province, China	—	—	JBDINY000000000
40	S39	—	Seawater from Fengjia Bay	2021	Wenchang city, Hainan Province, China	—	—	JBDINZ000000000
41	S40	—	Seawater from Fengjia Bay	2021	Wenchang city, Hainan Province, China	—	—	JBDIOA000000000
42	S41	—	Seawater from Hainan Bay	2022	Wenchang city, Hainan Province, China	—	—	JBDIOB000000000
43	S42	—	Seawater from Bamen Bay	2021	Wenchang city, Hainan Province, China	—	—	JBDIOC000000000

44	S43	—	Seawater from Hainan Bay	2022	Wenchang city, Hainan Province, China	—	—	JBDIOD000000000
45	SY1	—	Seawater from Phoenix Island	2023	Sanya city, Hainan Province, China	—	—	JBDIOE000000000
46	SY4	—	Seawater from Sanya Bay	2023	Sanya city, Hainan Province, China	—	—	JBDIOF000000000
47	SY5	—	Seawater from Sanya Bay	2023	Sanya city, Hainan Province, China	—	—	JBDIOG000000000
48	SY6	—	Seawater from Big East Sea Beach	2023	Sanya city, Hainan Province, China	—	—	JBDIOH000000000
49	SY9	—	Seawater from Big East Sea Beach	2023	Sanya city, Hainan Province, China	—	—	JBDIOI000000000
50	SY11	—	Seawater fromJiaotou Bay	2023	Sanya city, Hainan Province, China	—	—	JBDIOJ000000000
51	SY15	—	Seawater fromJiaotou Bay	2023	Sanya city, Hainan Province, China	—	—	JBDIOK000000000
52	SY17	—	Seawater from Yazhou Bay	2023	Sanya city, Hainan Province, China	—	—	JBDIOL000000000

53	SY101	—	Seawater from Sanya Bay	2023	Sanya city, Hainan Province, China	—	—	JBDIOM000000000
54	SY102	—	Seawater from Sanya Bay	2023	Sanya city, Hainan Province, China	—	—	JBDION000000000
55	SY103	—	Seawater from Sanya Bay	2023	Sanya city, Hainan Province, China	—	—	JBDIOO000000000
56	SY104	—	Seawater from Sanya Bay	2023	Sanya city, Hainan Province, China	—	—	JBDIOP000000000
57	HT103	—	Seawater from Hongtang Bay	2023	Sanya city, Hainan Province, China	—	—	JBDIOQ000000000
58	TH101	—	Seawater from Haitang Bay	2023	Sanya city, Hainan Province, China	—	—	JBDIOR000000000
59	XD101	—	Seawater from Little East Sea	2023	Sanya city, Hainan Province, China	—	—	JBDIOS000000000
60	YZ101	—	Seawater from Yazhou Bay	2023	Sanya city, Hainan Province, China	—	—	JBDIOT000000000
61	YZ102	—	Seawater from Yazhou Bay	2023	Sanya city, Hainan Province, China	—	—	JBDIOU000000000
62	YZ103	—	Seawater from Yazhou Bay	2023	Sanya city, Hainan Province, China	—	—	JBDIOV000000000
63	CCU101	—	Abscess	2013	China: Taiwan	Homo sapiens	Biliary Tract Diseases	GCA_003427415.1

64	KC-Na-R1	KCNaR1	Neophocaena phocaenoides	2017	South Korea: South sea	—	—	GCA_003721455.1
65	RQs-106	RQs106	Activated sludge	2011	China: Dalian	—	—	GCA_009730655.1
66	18064-CSB-B-B	CSBBB	Stool	2018	Tanzania	Poultry	—	GCA_009846595.1
67	2NE11	—	Olive production company	2018	Peru	—	—	GCA_014263185.1
68	VGH117	—	Tissue	2015	Taiwan	Homo sapiens	Infection	GCA_014702225.1
69	A291	—	Fish (cod)	1995	Denmark	—	—	GCA_017570045.1
70	A59	—	Flamingo	1980	France	—	—	GCA_017570125.1
71	G1	—	Feces	2016	Spain: Granada	—	—	GCA_017570225.1
72	150735	S150735	Human wound	2015	Spain: Gran Canaria	—	—	GCA_017570325.1
73	CECT 5071	CECT5071	—	1990	Japan	Jania sp. (Red alga)	—	GCA_009183365.2
74	ATCC 49138	ATCC49138	—	—	—	—	—	GCA_019670525.1
75	TUM4442	—	—	—	Japan:Tokyo	Homo sapiens	—	GCA_019670545.1
76	TUM17378	—	—	2014	Japan:Tokyo	Homo sapiens	—	GCA_019670585.1

77	TUM17379	—	—	2014	Japan:Tokyo	Homo sapiens	—	GCA_019670605.1
78	TUM17382	—	—	2015	Japan:Tokyo	Homo sapiens	—	GCA_019670625.1
79	TUM17383	—	—	2015	Japan:Tokyo	Homo sapiens	—	GCA_019670645.1
80	TUM17384	—	—	2015	Japan:Tokyo	Homo sapiens	—	GCA_019670665.1
81	TUM17386	—	—	2015	Japan:Tokyo	Homo sapiens	—	GCA_019670685.1
82	VGH117	VGH1171	Blood	2015	Taiwan	Homo sapiens	Infection	GCA_025643615.1
83	B2215466	—	Patient Blood	2022	Germany: Marburg	Homo sapiens	Sepsis	GCA_026013945.1
84	MCCC 1A11468	MCCC1A1146 8	Seawater	2017	China: South China Sea, Guangdong	—	—	GCA_030436035.1
85	EBI	—	Ciliate: Metopus sp. strain NARRIV	2021	USA	Metopus sp. strain NARRIV	—	GCA_963674875.1
86	CCU4051	—	—	2016	Taiwan	Homo sapiens	Biliary tract disease	GCA_019264685.1

87	CCU4052	—	—	2016	Taiwan	Homo sapiens	Biliary tract disease	GCA_019265005.1
88	CCU4053	—	—	2016	Taiwan	Homo sapiens	Biliary tract disease	GCA_019265025.1
89	CCU4054	—	—	2016	Taiwan	Homo sapiens	Biliary tract disease	GCA_019265045.1
90	NCTC10738	—	Stool	1970	—	—	—	GCA_900457125.1
91	MARS 14	—	—	—	—	—	—	GCA_000947195.1
92	CCUG-48086	CCUG48086	Blood	2003	Sweden	—	—	GCA_017580505.1
93	LZ201228	—	Stool	2012	China: Shandong	Homo sapiens	—	GCA_029750685.1
94	AC	—	Haliotis diversicolor	2014	Taiwan	—	—	GCA_003024535.1
95	CCUG-38646	CCUG38646	Blood	1997	Norway	Homo sapiens	—	GCA_017580485.1
96	LZ201228	LZ2012281	—	2012	China:Laizhou	Homo sapiens	—	GCA_019303305.1
97	YHL	—	Wound	2014	Taiwan	Homo sapiens	Wound infection	GCA_002318995.1
98	HUD-D8	HUDD8	Blood	2016	Sweden	Homo sapiens	—	GCA_017579985.1
99	669801	S669801	Wound	2016	Spain: Gran Canaria	Homo sapiens	—	GCA_017580325.1
100	A60	—	—	1980	France	flamingo	—	GCA_017580095.1

101	CCUG-56496	CCUG56496	Trachea	2008	Sweden	Homo sapiens	—	GCA_017580455.1
102	CLS4	—	Blood	2014	Taiwan	Homo sapiens	Bacteremia	GCA_007595165.1
103	SYT2	—	Crassostrea gigas	2014	China: Taiwan	—	—	GCA_007595325.1
104	CCUG-12945	CCUG12945	Wound	1982	USA	Homo sapiens	—	GCA_017580665.1
105	SYT4	—	Meretrix lusoria	2014	China: Taiwan	—	—	GCA_003024575.1
106	C6G3	—	Marine sediment	2007	France: Arcahon Bay	—	—	GCA_000956365.1
107	A94	—	Blood	1994	Denmark	Homo sapiens	—	GCA_017580105.1
108	JFC3	—	Crassostrea gigas	2014	China: Taiwan	—	—	GCA_007636355.1
109	JFL	—	Blood	2014	China: Taiwan	Homo sapiens	Bacteremia	GCA_007636455.1
110	CCUG-526	CCUG526	Ear	1969	Sweden	Homo sapiens	—	GCA_017580685.1
111	A292	—	—	1995	Denmark	Fish	—	GCA_017580235.1
112	950570	S950570	Feces	2019	Spain: Granada	Homo sapiens	—	GCA_017580295.1

113	CCUG-72678	CCUG72678	Crushwound	2018	Sweden	Homo sapiens	—	GCA_017580415.1
114	JFC2	—	Crassostrea gigas	2014	China: Taiwan	—	—	GCA_007844895.1
115	M69b	—	—	2021	Not applicable	—	—	GCA_028594625.1
116	CLS5	—	Bile	2014	China: Taiwan	Homo sapiens	cholecytitis	GCA_007595175.1
117	CCUG-15259	CCUG15259	Ear	1984	USA	Homo sapiens	—	GCA_017580585.1
118	SF7	—	Urinary catheter exudate	2010	Spain: Gran Canaria	Homo sapiens	—	GCA_017580025.1
119	A56	—	—	1980	Senegal	Fish	—	GCA_017580215.1
120	CCUG-58400	CCUG58400	Intestine	2009	South Korea	Upeneus japonicus	—	GCA_017580385.1
121	melkephyllucas	melkephy	Blood	2014	China: Taiwan	Homo sapiens	Bacteremia	GCA_007595215.1
122	ATCC 51192	ATCC51192	—	—	France: Coast of Arcachon Bay	Red alga	—	GCA_012396675.1
123	5043	S5043	Wound	2015	Spain: Gran Canaria	Homo sapiens	—	GCA_017580635.1
124	SYC	—	Blood	2014	China: Taiwan	Homo sapiens	Bacteremia	GCA_008119825.1

125	08MAS2314	MAS2314	Stool	2008	China: Anhui	Homo sapiens	—	GCA_029750625.1
126	20-23R	S2023R	Intestine	—	South Korea: South Sea	Upeneus japonicus	—	GCA_002836995.1
127	CHL	—	Bile	2014	China: Taiwan	Homo sapiens	Cholecytitis	GCA_007595205.1
128	RC	—	Blood	2014	China: Taiwan	Homo sapiens	Bacteremia	GCA_007636395.1
129	CCUG-20533	CCUG20533	Sputum	1987	Sweden	Homo sapiens	—	GCA_017580595.1
130	08MAS2314	MAS23142	—	2008	China:Ma'anshan	Homo sapiens	—	GCA_019303435.1
131	YTH	—	Blood	2014	China: Taiwan	Homo sapiens	Bacteremia	GCA_007636365.1
132	HUD-H4	HUDH4	wound	2018	Sweden	Homo sapiens	—	GCA_017580015.1
133	CCUG-72638	CCUG72638	Blood	2018	Sweden	Homo sapiens	—	GCA_017580425.1
134	HUD-I2	HUDI2	Wound	2018	Sweden	Homo sapiens	—	GCA_017580005.1
135	A65	—	—	1982	France	poultry	—	GCA_017580125.1
136	HN_2022	HN2022	Infection secretions from patient	2022	China:Hainan	Homo sapiens	—	GCA_025398225.1

137	LC4	—	—	2015	Brazil: Buzios, Rio de Janeiro	Siderastrea stellata	—	GCA_030264795.1
138	A97	—	Wound	1992	Denmark	Homo sapiens	—	GCA_017580085.1
139	159418	S159418	Ulcer	2015	Spain: Gran Canaria	Homo sapiens	—	GCA_017580345.1
140	CCUG-789	CCUG789	Infection	1970	USA	Homo sapiens	—	GCA_017580705.1
141	254-1	S2541	Bile	2014	Spain: Gran Canaria	Homo sapiens	—	GCA_023283525.1
142	YK-SH	YKSH	Rearing water	2020	China: Liaoning	—	—	GCA_036181485.1
143	SYCDC19SW06	DC19SW06	Fecal sample from patient with diarrhea	2019	China: Beijing	Homo sapiens	Acute diarrhea	GCA_037006705.1
144	A93	—	Blood	1994	Denmark	Homo sapiens	—	GCA_017580145.1
145	YTL	—	Blood	2014	China: Taiwan	Homo sapiens	Bacteremia	GCA_007636415.1
146	SYCDC18SW04	DC18SW04	Fecal sample from patient with diarrhea	2018	China: Beijing	Homo sapiens	Acute diarrhea	GCA_037006925.1

147	SYCDC19SW05	DC19SW05	Fecal sample from patient with diarrhea	2019	China: Beijing	Homo sapiens	Acute diarrhea	GCA_037006745.1
148	SYCDC19SW01	DC19SW01	Fecal sample from patient with diarrhea	2019	China: Beijing	Homo sapiens	Acute diarrhea	GCA_037006825.1
149	HUD-G3	HUDG3	Blood	2018	Sweden	Homo sapiens	—	GCA_017580065.1
150	SYCDC17SW07	DC17SW07	Fecal sample from patient with diarrhea	2017	China: Beijing	Homo sapiens	Acute diarrhea	GCA_037007105.1
151	6F5	S6F5	Bronchial aspirate	2011	Spain: Gran Canaria	—	—	GCA_017580285.1
152	CCUG-50501	CCUG50501	Wound	2010	Sweden	Homo sapiens	—	GCA_017580535.1
153	28011	S28011	Wound	2014	Spain: Gran Canaria	Homo sapiens	—	GCA_017580565.1
154	404	S404	cutaneous biopsy	2008	Spain: Gran Canaria	Homo sapiens	—	GCA_017580715.1
155	JC874	—	—	2022	India:Pudumadam	Green algae	—	GCA_032843145.1
156	CSB04KR	—	Gut	2015	South Korea: Geomun-do	Apostichopus japonicus	—	GCA_001858195.1
157	590722	S590722	Ear	2018	Spain: Gran Canaria	Homo sapiens	—	GCA_017580335.1

158	OTH-19-VL-WA-NY-0079	OTH19	Skin (Canthigaster valentini)	2019	USA:NY	—	—	GCA_029771985.1
159	ACCC	—	Bile	2014	China: Taiwan	Homo sapiens	Cholecytitis	GCA_003025175.1
160	SYCDC18SW07	DC18SW07	Fecal sample from patient with diarrhea	2018	China: Beijing	Homo sapiens	Acute diarrhea	GCA_037006905.1
161	LC2016-5	LC201651	Blood	2016	China: Shandong	Homo sapiens	Patient with bacteremia	GCA_029750645.1
162	LC2016-5	LC201652	—	2016	China:Laizhou	Homo sapiens	—	GCA_019303345.1
163	SYCDC18SW10	DC18SW10	Fecal sample from patient with diarrhea	2018	China: Beijing	Homo sapiens	Acute diarrhea	GCA_037006865.1
164	SYCDC17SW02	DC17SW02	Fecal sample from patient with diarrhea	2017	China: Beijing	Homo sapiens	Acute diarrhea	GCA_037007185.1
165	B29	—	—	2022	Saudi Arabia	—	—	GCA_023702245.1
166	LCU-VS1	LCUVS1	Tissue sample	2022	China: Shandong	Penaeus vannamei	—	GCA_030646225.1
167	CCUG-24987	CCUG24987	Blood	1989	Sweden	Homo sapiens	—	GCA_017580525.1

168	SYCDC17SW03	DC17SW03	Fecal sample from patient with diarrhea	2017	China: Beijing	Homo sapiens	Acute diarrhea	GCA_037007145.1
169	LZ2015243	—	—	2015	China:Laizhou	Homo sapiens	—	GCA_019303235.1
170	SYCDC17SW04	DC17SW04	Fecal sample from patient with diarrhea	2017	China: Beijing	Homo sapiens	Acute diarrhea	GCA_037007125.1
171	JFC1	—	—	2014	China: Taiwan	Magallana gigas	—	GCA_007860205.1
172	SYCDC19SW04	DC19SW04	Fecal sample from patient with diarrhea	2019	China: Beijing	Homo sapiens	Acute diarrhea	GCA_037006765.1
173	A57	—	Wound	1980	France	Homo sapiens	—	GCA_017580185.1
174	LZ2013652	—	—	2013	China:Laizhou	Homo sapiens	—	GCA_019303255.1
175	97087	S97087	Wound	2015	Spain: Gran Canaria	Homo sapiens	—	GCA_017580395.1
176	TYL	—	Blood	2014	China: Taiwan	Homo sapiens	Bacteremia	GCA_007636495.1
177	SYCDC17SW01	DC17SW01	Fecal sample from patient with diarrhea	2017	China: Beijing	Homo sapiens	Acute diarrhea	GCA_037007205.1

178	SYT1	—	—	2014	China: Taiwan	Haliotis diversicolor	—	GCA_007595095.1
179	SYT3	—	Ocean water	2014	China: Taiwan	—	—	GCA_007595405.1
180	SYCDC17SW05	DC17SW05	Fecal sample from patient with diarrhea	2017	China: Beijing	Homo sapiens	Acute diarrhea	GCA_037007155.1
181	LC2016-6	LC20166	—	2016	China:Laizhou	Homo sapiens	—	GCA_019303385.1
182	JCM 21037	JCM21037	—	—	—	—	—	GCA_000615045.1
183	SYCDC17SW06	DC17SW06	Fecal sample from patient with diarrhea	2017	China: Beijing	Homo sapiens	Acute diarrhea	GCA_037007065.1
184	A58	—	—	1980	France	Flamingo	—	GCA_017580205.1
185	MAS2736	MAS27361	—	2007	China:Ma'anshan	Homo sapiens	—	GCA_019303295.1
186	CLS2	—	Blood	2014	China: Taiwan	Homo sapiens	Bacteremia	GCA_007595365.1
187	SYCDC18SW03	DC18SW03	Fecal sample from patient with diarrhea	2018	China: Beijing	Homo sapiens	Acute diarrhea	GCA_037007005.1
188	SYCDC17SW08	DC17SW08	Fecal sample from patient with diarrhea	2017	China: Beijing	Homo sapiens	Acute diarrhea	GCA_037007085.1

189	SYCDC18SW09	DC18SW09	Fecal sample from patient with diarrhea	2018	China: Beijing	Homo sapiens	Acute diarrhea	GCA_037006845.1
190	LC2016-4	LC20164	—	2016	China:Laizhou	Homo sapiens	—	GCA_019303335.1
191	LC2016-1	LC20161	—	2016	China:Laizhou	Homo sapiens	—	GCA_019303455.1
192	JCM 14758	JCM14758	—	—	—	—	—	GCA_000614935.1
193	MN-01	MN01	Salt marsh sediment	2010	USA:GA, Skidaway Island	—	—	GCA_002237105.1
194	A41	—	Feces	1963	Japan	Homo sapiens	—	GCA_017580225.1
195	INSAq258	—	Mussels	2019	Portugal	—	—	GCA_021441365.1
196	INSAq494	—	Mussels	2019	Portugal	—	—	GCA_021440145.1
197	NBRC 103173	NBRC1031	—	—	—	—	—	GCA_001598875.1
198	MAS2736	—	Water	2010	China:Beijing	—	—	GCA_029750665.1
199	Sh392	—	Skin and soft tissue infection	2006	Argentina:Buenos Aires	Homo sapiens	Skin and soft tissue infection	GCA_003124085.1
200	LC2016-2	LC20162	—	2016	China:Laizhou	Homo sapiens	—	GCA_019303415.1
201	INSAq495	—	Mussels	2019	Portugal	—	—	GCA_021440725.1

202	CLS1	—	Wound	2014	China: Taiwan	Homo sapiens	Wound infection	GCA_007595085.1
203	LC2016-3	LC20163	—	2016	China:Laizhou	Homo sapiens	—	GCA_019303375.1
204	LZ2015256	—	—	2015	China:Laizhou	Homo sapiens	—	GCA_019303275.1
205	SYCDC18SW01	DC18SW01	Fecal sample from patient with diarrhea	2018	China: Beijing	Homo sapiens	Acute diarrhea	GCA_037007025.1
206	CLS3	—	Blood	2014	China: Taiwan	Homo sapiens	Bacteremia	GCA_007595375.1

The No. 1-62 strains are isolated in this study.

Table S2. Detailed information of 206 *S. algae* genomes

NO.	Renamed strain	Contigs /Scaffolds	Total Length(bp)	N50 Length(bp)	Max Length(bp)	Min Length(bp)	G+C (mol %)	Gene number	Number of tRNA
1	12	46	4,828,472	205,267	423,880	658	53.01	4,339	100
2	13	44	4,917,425	244,507	577,591	522	53	4,423	104
3	14	40	4,976,312	237,553	603,506	580	53.02	4,503	94
4	2NE11	1	5,030,813	5,030,813	5,030,813	5,030,813	52.98	4,518	112
5	5	46	4,827,936	205,620	423,880	678	53.01	4,338	100
6	6638	38	4,865,967	285,893	433,504	2,401	53.06	4,383	99
7	6	85	4,686,332	127,492	297,631	839	53.15	4,283	105

8	A291	1	4,971,565	4,971,565	4,971,565	4,971,565	52.97	4,421	105
9	A292	46	4,957,768	292,820	669,424	558	53.02	4,487	93
10	A41	129	4,869,205	100,124	204,690	506	52.9	4,426	96
11	A56	51	4,848,386	224,144	532,373	516	53.02	4,380	93
12	A57	95	4,660,999	117,839	305,175	542	53.15	4,251	97
13	A58	111	4,822,783	92,016	603,602	537	53.01	4,363	91
14	A59	1	4,817,651	4,817,651	4,817,651	4,817,651	53.1	4,316	108
15	A60	38	4,818,514	302,913	763,184	503	52.95	4,325	98
16	A65	61	4,901,782	188,949	719,281	507	53.09	4,422	92
17	A93	64	4,957,397	163,674	1,050,398	506	52.91	4,470	96
18	A94	44	4,882,077	408,692	1,345,816	547	53.04	4,432	93
19	A97	62	5,005,946	198,761	471,058	549	52.95	4,554	92
20	ACCC	74	4,744,804	118,624	589,495	1,017	53.08	4,307	92
21	AC	28	4,753,352	413,405	1,163,829	1,160	53.09	4,297	86
22	ATCC49138	1	4,849,690	4,849,690	4,849,690	4,849,690	52.97	4,431	104
23	ATCC51192	52	4,978,360	224,036	1,051,072	1,149	52.49	4,498	95
24	B2215466	1	4,946,841	4,946,841	4,946,841	4,946,841	52.94	4,464	106
25	B29	74	4,826,109	151,956	387,929	500	53.05	4,359	94
26	C6G3	43	4,879,425	364,658	498,365	858	53.08	5,917	86
27	CCU101	2	4,919,337	4,786,791	4,786,791	132,546	53.04	4,806	113
28	CCU4051	2	4,919,537	4,786,891	4,786,891	132,646	53.04	4,806	113
29	CCU4052	2	4,919,930	4,787,275	4,787,275	132,655	53.04	4,587	113
30	CCU4053	2	4,919,868	4,787,217	4,787,217	132,651	53.04	4,596	112
31	CCU4054	2	4,919,717	4,787,071	4,787,071	132,646	53.04	4,676	113
32	CCUG12945	40	4,875,886	266,947	1,246,063	501	52.98	4,392	95
33	CCUG15259	50	4,891,496	287,248	1,074,525	545	52.97	4,425	89

34	CCUG20533	58	4,835,934	165,595	662,143	569	52.98	4,365	93
35	CCUG24987	89	4,814,300	127,122	311,183	503	52.95	4,367	90
36	CCUG38646	35	4,790,201	443,518	911,909	543	53.05	4,315	94
37	CCUG48086	33	4,844,414	462,889	1,412,155	509	53.04	4,363	91
38	CCUG50501	71	5,016,616	392,359	1,255,113	511	52.76	4,524	94
39	CCUG526	45	4,784,369	358,290	873,537	516	53.11	4,324	92
40	CCUG56496	38	4,796,178	271,375	1,022,198	501	53.14	4,339	96
41	CCUG58400	51	4,766,857	249,418	590,141	513	53.07	4,282	97
42	CCUG72638	60	4,876,138	263,069	677,495	515	53.04	4,346	94
43	CCUG72678	46	4,870,233	328,667	848,855	504	53	4,376	98
44	CCUG789	62	4,863,341	175,015	621,130	564	53.06	4,415	95
45	CECT5071	1	4,924,764	4,924,764	4,924,764	4,924,764	53.05	4,434	107
46	CHL	55	4,888,589	162,092	429,624	1,319	52.96	4,441	89
47	CLS1	159	4,887,246	70,370	217,863	1,006	53.03	4,456	91
48	CLS2	115	4,878,731	78,097	281,057	1,031	52.93	4,365	89
49	CLS3	128	4,603,128	92,159	222,936	524	53.08	4,193	82
50	CLS4	35	4,866,748	223,458	1,200,651	1,149	53.03	4,483	88
51	CLS5	43	4,824,095	194,183	591,209	1,230	52.98	4,392	93
52	CSB04KR	64	4,803,356	149,689	301,346	565	53.03	4,361	92
53	CSBBB	2	4,903,874	4,754,321	4,754,321	149,553	53.03	4,476	108
54	DC17SW01	102	4,717,715	85,432	279,700	525	53.08	4,278	54
55	DC17SW02	87	4,762,367	120,519	223,489	515	53.05	4,323	48
56	DC17SW03	89	4,757,243	120,527	241,182	525	53.05	4,324	41
57	DC17SW04	90	4,759,249	120,527	223,489	515	53.05	4,329	43
58	DC17SW05	103	4,676,070	86,050	212,551	615	53.06	4,239	47
59	DC17SW06	107	4,676,682	82,131	248,934	615	53.05	4,239	52

60	DC17SW07	68	4,869,715	132,727	421,337	531	53.11	4,436	59
61	DC17SW08	115	4,888,461	117,620	239,976	502	52.94	4,382	44
62	DC18SW01	162	4,620,397	54,299	172,796	524	53.01	4,207	56
63	DC18SW03	114	4,755,113	92,255	342,616	519	52.99	4,322	48
64	DC18SW04	66	4,821,134	171,565	461,357	541	53.1	4,367	46
65	DC18SW07	81	4,638,041	122,798	309,680	501	53.15	4,203	59
66	DC18SW09	117	4,677,420	97,525	278,046	507	53.17	4,238	46
67	DC18SW10	87	4,676,695	105,496	299,239	512	53.15	4,241	56
68	DC19SW01	68	4,763,884	134,666	522,481	551	53.04	4,263	49
69	DC19SW04	93	4,819,358	96,970	304,429	517	52.97	4,336	46
70	DC19SW05	67	4,875,902	151,568	486,431	567	52.91	4,410	51
71	DC19SW06	63	4,876,409	189,850	415,645	793	52.91	4,415	47
72	EBI	1	4,824,153	4,824,153	4,824,153	4,824,153	53.18	4,338	106
73	G1	1	4,887,746	4,887,746	4,887,746	4,887,746	52.97	4,413	106
74	HN2022	57	4,797,750	180,791	368,353	524	53.09	4,297	96
75	HT103	53	4,860,262	209,508	737,553	550	53.09	4,349	101
76	HUDD8	36	4,831,989	226,833	860,533	536	53.07	4,356	93
77	HUDG3	69	4,980,582	169,739	488,211	545	52.75	4,483	94
78	HUDH4	60	4,932,276	267,638	605,993	514	52.93	4,474	100
79	HUDI2	61	4,932,981	267,638	605,999	505	52.93	4,477	98
80	INSAq258	94	4,816,413	131,443	401,660	546	53.06	4,304	83
81	INSAq494	92	4,856,586	120,133	418,135	583	53.06	4,347	73
82	INSAq495	110	4,811,418	119,234	280,581	507	53.06	4,300	70
83	JC874	48	4,832,296	305,464	1,228,541	514	53	4,340	96
84	JCM14758	125	4,929,916	74,366	233,475	511	52.9	5,553	60
85	JCM21037	108	4,827,686	103,016	305,083	537	53.04	5,290	65

86	JFC1	91	4,803,344	98,142	269,396	1,001	53.01	4,371	96
87	JFC2	44	4,840,125	202,873	1,034,750	1,049	53.03	4,341	88
88	JFC3	43	4,819,646	220,614	662,381	1,346	53	4,322	91
89	JFL	41	4,801,112	264,829	1,115,304	1,336	53.11	4,347	92
90	KCNaR1	2	5,203,444	5,036,300	5,036,300	167,144	52.79	4,737	103
91	LC20161	120	4,680,828	87,342	282,758	1,005	53.08	4,264	112
92	LC20162	155	4,661,397	66,841	203,209	507	53.09	4,250	49
93	LC20163	157	4,658,855	67,674	166,246	507	53.09	4,249	50
94	LC20164	117	4,676,305	87,235	282,777	1,025	53.09	4,262	112
95	LC201651	81	4,939,669	128,312	255,862	584	52.95	4,533	94
96	LC201652	87	4,857,637	118,094	256,215	523	52.94	4,490	110
97	LC20166	105	4,843,187	98,511	223,179	501	52.93	4,474	45
98	LC4	47	5,011,050	273,138	718,945	522	53.04	4,480	116
99	LCUVS1	32	4,778,864	313,698	597,447	536	53.16	4,334	103
100	LZ2012281	34	4,868,151	366,387	773,111	507	53.11	4,405	112
101	LZ201228	31	4,938,237	440,032	1,136,626	603	53.1	4,472	98
102	LZ2013652	94	4,708,546	100,763	288,096	521	53.1	4,296	56
103	LZ2015243	89	4,803,486	97,980	419,980	533	53.03	4,329	95
104	LZ2015256	160	4,769,617	63,613	216,271	568	53.03	4,205	99
105	M69b	40	4,865,598	333,833	1,060,796	593	52.93	4,381	97
106	MARS	11	5,005,849	4,590,164	4,590,164	804	52.88	4,460	105
107	MAS23142	57	4,792,513	183,772	438,576	508	53.07	4,313	99
108	MAS2314	55	4,851,061	260,707	488,510	502	53.08	4,351	97
109	MAS27361	38	4,810,933	1,411,445	1,445,047	515	53.03	4,319	94
110	MAS2736	149	4,765,437	58,095	238,547	503	53.07	4,301	31

111	MCCC1A11468	1	4,880,117	4,880,117	4,880,117	4,880,117	53.1	4,362	110
112	melkephy	51	4,808,216	209,797	577,344	1,056	53.05	4,360	82
113	MN01	102	4,765,126	96,683	284,248	806	53.02	4,264	69
114	NBRC1031	143	4,817,247	65,053	155,184	520	53.06	4,341	59
115	NCTC10738	3	4,997,042	3,159,547	3,159,547	8,194	52.83	4,556	108
116	OTH19	69	4,959,286	140,744	471,076	506	52.9	4,504	96
117	RC	54	4,807,239	200,902	662,666	1,304	53.03	4,312	88
118	RQs106	1	4,990,025	4,990,025	4,990,025	4,990,025	52.95	4,466	106
119	S10	69	4,868,258	164,121	313,127	818	53.02	4,365	106
120	S11	70	4,866,522	170,850	365,994	546	53.03	4,361	97
121	S12	47	5,035,775	272,442	1,051,681	820	52.99	4,621	98
122	S13	40	4,815,394	189,884	683,765	801	53.06	4,306	105
123	S14	42	4,764,266	281,030	721,039	990	53.11	4,288	106
124	S150735	1	5,070,545	5,070,545	5,070,545	5,070,545	52.81	4,569	108
125	S159418	62	4,769,540	183,110	719,183	500	52.96	4,300	98
126	S15	35	4,879,179	452,083	777,223	513	53.11	4,394	99
127	S16	75	4,788,632	161,453	389,819	518	53.09	4,331	99
128	S17	42	4,815,466	189,884	683,659	801	53.06	4,304	101
129	S18	46	4,818,919	293,200	536,621	554	53.05	4,401	103
130	S19	55	5,002,553	187,112	575,393	554	52.9	4,587	99
131	S1	64	4,847,814	169,262	397,330	628	53	4,390	100
132	S2023R	56	4,758,780	165,375	487,435	1,138	53.08	4,271	113
133	S20	65	4,962,015	170,245	409,183	502	53.04	4,495	99
134	S21	74	4,817,340	155,940	321,220	501	52.99	4,331	102
135	S22	39	4,923,823	240,880	825,833	761	52.89	4,468	101

136	S24	83	4,899,458	142,710	343,988	529	52.96	4,429	105
137	S2541	62	4,763,835	171,431	418,142	542	52.96	4,291	94
138	S26	98	4,872,346	101,496	338,301	531	52.96	4,393	95
139	S27	46	4,917,152	227,503	932,081	524	53.06	4,395	99
140	S28011	71	4,943,711	168,966	540,700	510	53	4,488	95
141	S28	44	4,887,904	374,024	515,395	533	52.94	4,361	100
142	S2	50	4,839,085	290,675	997,177	581	53.1	4,393	104
143	S31	63	4,949,390	160,333	476,633	553	52.89	4,458	101
144	S32	64	4,834,362	192,915	613,726	694	52.86	4,351	104
145	S33	46	4,861,687	195,512	534,966	745	52.98	4,435	100
146	S34	36	4,852,418	351,500	1,179,088	537	53.01	4,413	96
147	S35	72	5,004,734	161,274	446,323	555	52.89	4,594	103
148	S36	18	5,097,532	415,572	1,055,313	50,946	53.15	4,629	113
149	S37	32	4,969,505	355,339	760,572	1,198	53.09	4,480	93
150	S39	49	4,829,189	190,360	522,732	592	52.89	4,374	102
151	S3	49	4,840,483	229,187	997,875	633	53.1	4,387	106
152	S404	71	4,880,812	181,943	419,683	560	53.08	4,418	93
153	S40	53	5,069,133	224,764	414,505	611	52.93	4,589	103
154	S41	53	4,805,524	178,202	721,233	819	53.01	4,319	108
155	S42	53	4,904,171	189,802	602,215	536	53	4,414	98
156	S43	35	4,807,432	349,412	1,249,344	571	53.04	4,333	96
157	S4	48	4,837,864	229,151	997,757	633	53.1	4,387	104
158	S5043	52	4,997,180	362,228	534,620	547	52.95	4,557	84
159	S590722	74	4,992,475	146,526	548,913	503	52.91	4,490	95
160	S5	50	4,839,345	229,151	998,066	633	53.1	4,390	106
161	S669801	36	4,776,174	377,824	1,423,946	530	53.14	4,290	88

162	S6F5	71	4,870,277	207,082	363,011	543	53	4,398	97
163	S6	67	4,870,459	161,259	539,971	516	53.01	4,372	100
164	S7	35	4,952,528	367,574	853,026	520	53.07	4,525	98
165	S8	49	4,968,144	239,976	713,672	773	52.89	4,504	106
166	S950570	46	4,756,369	268,654	1,050,172	529	53.14	4,263	100
167	S97087	98	4,856,502	132,356	367,416	510	52.91	4,392	98
168	S9	76	4,866,873	139,532	303,021	796	53.03	4,357	103
169	SF7	51	4,843,605	302,614	875,358	536	53.01	4,385	93
170	Sh392	107	4,778,190	96,236	367,520	514	52.94	4,321	107
171	SY101	105	5,054,536	116,235	250,435	544	52.72	4,601	101
172	SY102	81	5,107,245	162,652	307,093	623	52.74	4,676	100
173	SY103	47	4,782,351	229,420	572,178	523	53.15	4,350	98
174	SY104	40	4,782,630	321,720	746,592	693	53.04	4,301	96
175	SY11	58	4,993,056	203,805	439,187	515	53.04	4,516	107
176	SY15	44	4,890,582	204,626	628,463	523	53.1	4,436	98
177	SY17	60	4,919,560	184,017	519,657	527	52.95	4,399	102
178	SY1	67	4,788,626	158,554	564,242	585	53.05	4,359	99
179	SY4	52	4,826,079	171,112	494,180	584	53.09	4,385	97
180	SY5	49	4,933,071	278,003	786,229	683	52.98	4,441	101
181	SY6	24	4,795,759	458,324	808,912	888	53.14	4,314	107
182	SY9	24	4,795,820	741,217	880,617	1,023	53.14	4,317	106
183	SYC	49	4,770,716	171,304	466,763	1,102	52.94	4,309	82
184	SYT1	103	4,814,598	108,667	309,963	1,037	53.09	4,329	89
185	SYT2	37	4,782,532	295,771	685,673	1,123	53.1	4,288	84
186	SYT3	80	4,823,421	341,750	1,158,312	502	53	4,365	77
187	SYT4	37	4,839,649	328,146	884,367	1,100	53.09	4,333	91

188	TH101	59	4,867,391	207,474	419,825	500	53.03	4,380	99
189	TUM17378	1	4,872,739	4,872,739	4,872,739	4,872,739	52.97	4,365	107
190	TUM17379	1	4,909,921	4,909,921	4,909,921	4,909,921	52.98	4,437	105
191	TUM17382	1	5,040,456	5,040,456	5,040,456	5,040,456	52.93	4,545	103
192	TUM17383	1	4,921,266	4,921,266	4,921,266	4,921,266	53.09	4,393	102
193	TUM17384	1	4,871,625	4,871,625	4,871,625	4,871,625	53.07	4,367	103
194	TUM17386	1	4,911,443	4,911,443	4,911,443	4,911,443	53.07	4,400	104
195	TUM4442	1	4,798,767	4,798,767	4,798,767	4,798,767	53.13	4,275	106
196	TYL	100	4,821,720	96,168	234,074	1,122	52.95	4,344	91
197	VGH1171	2	4,919,668	4,787,117	4,787,117	132,551	53.04	4,596	112
198	VGH117	1	4,796,801	4,796,801	4,796,801	4,796,801	53.14	4,307	106
199	XD101	20	4,809,236	451,743	807,195	33,575	53.11	4,354	95
200	YHL	27	4,850,439	357,371	976,090	1,367	53	4,352	87
201	YKSH	58	4,944,863	179,302	897,916	538	53	4,444	93
202	YTH	52	4,811,827	179,489	662,669	1,126	53.03	4,322	88
203	YTL	60	4,785,826	155,918	519,243	1,000	52.91	4,328	82
204	YZ101	49	4,782,982	190,483	347,197	682	53.08	4,314	100
205	YZ102	49	4,780,811	176,436	438,064	523	53.08	4,313	99
206	YZ103	44	4,778,534	249,117	346,907	508	53.09	4,306	93

Table S3. The classification of virulence related factors of *S. algae* against VFDB

**VF iD**	**Cluster**	**Virulence factor**	**VF category**
AI097	C1	Type IV pili	Adherence; Fimbrial adhesin; Type IV pili; Type IVa pili (T4aP)
AI103	C1	Tap pili	Adherence; Fimbrial adhesin; Type IV pili; Type IVa pili (T4aP)
AI111	C1	Type IV pili	Adherence; Fimbrial adhesin; Type IV pili; Type IVa pili (T4aP)

AI117	C1	Type IV pili	Adherence; Fimbrial adhesin; Type IV pili; Type IVa pili (T4aP)
AI118	C1	Type IV pili	Adherence; Fimbrial adhesin; Type IV pili; Type IVa pili (T4aP)
AI138	C1	Flagella	Motility; Flagella-mediated motility
AI140	C1	Peritrichous flagella	Motility; Flagella-mediated motility
AI142	C1	lateral flagella	Motility
AI143	C1	Lateral flagella	Motility; Flagella-mediated motility
AI144	C1	Polar flagella	Motility; Flagella-mediated motility
AI145	C1	Peritrichous flagella	Motility; Flagella-mediated motility
AI149	C1	Polar flagella	Motility; Flagella-mediated motility; Flagella assembly
AI227	C1	Fibronectin-binding protein	Adherence
AI331	C1	NlpI	Post-translational modification
AI361	C1	MG1142	Adherence; binds to heparin
AI392	C1	Hsp70	Adherence
CVF010	C1	PhoPQ	Regulation
CVF123	C1	Streptococcal plasmin receptor/GAPDH	Adherence
CVF153	C1	Streptococcal enolase	Exoenzyme
CVF171	C1	Beta-hemolysin/cytolysin	Exotoxin
CVF186	C1	Capsule	Immune modulation
CVF228	C1	Listeria adhesion protein	Adherence; Non-fimbrial adhesin; Cell wall anchored protein
CVF259	C1	Mannose-sensitive hemagglutinin (MSHA type IV pilus)	Adherence; Fimbrial adhesin; Type IV pili; Type IVa pili (T4aP)
CVF269	C1	VAS T6SS	Effector delivery system; Type VI secretion system (T6SS); T6SS components
CVF270	C1	VAS T6SS	Effector delivery system; Type VI secretion system (T6SS); T6SS components
CVF276	C1	ViuPDGC system	Nutritional/Metabolic factor

CVF277	C1	Enterobactin receptors	Nutritional/Metabolic factor
CVF281	C1	Flagella	Motility; Flagella-mediated motility
CVF282	C1	Capsular polysaccharide	Immune modulation; Antiphagocytosis
CVF300	C1	FAS-II	Nutritional/Metabolic factor
CVF302	C1	Isocitrate lyase	Others
CVF305	C1	Pantothenate synthesis	Nutritional/Metabolic factor; Metabolic adaptation
CVF308	C1	Tryptophan synthesis	Nutritional/Metabolic factor
CVF309	C1	Leucine synthesis	Nutritional/Metabolic factor
CVF311	C1	Glutamine synthesis	Nutritional/Metabolic factor
CVF322	C1	AhpC	Stress survival
CVF332	C1	PrrA/B	Regulation
CVF333	C1	MprA/B	Regulation
CVF347	C1	Hsp60	Adherence; Non-fimbrial adhesin; Cell wall anchored protein
CVF349	C1	Mip	Post-translational modification
CVF358	C1	Cytochrome c maturation (ccm) locus	Nutritional/Metabolic factor; Metal uptake; Iron uptake; Siderophore uptake system; Siderophore biosynthesis
CVF359	C1	Ferrous iron transport	Nutritional/Metabolic factor; Metal uptake; Iron uptake; Ferrous iron binding
CVF362	C1	Carbon storage regulator A	Regulation
CVF365	C1	RelA	Regulation
CVF383	C1	LPS	Immune modulation; Inflammatory signaling pathway
CVF393	C1	Capsule biosynthesis and transport	Immune modulation; Antiphagocytosis
CVF396	C1	LOS	Immune modulation; Inflammatory signaling pathway
CVF399	C1	O-linked flagellar glycosylation	Motility
CVF460	C1	Chu	Nutritional/Metabolic factor; Metal uptake; Iron uptake; Heme uptake system

CVF478	C1	Enterobactin transport	Nutritional/Metabolic factor; Metal uptake; Iron uptake; Siderophore uptake system
CVF494	C1	LOS	Immune modulation; Inflammatory signaling pathway
CVF495	C1	Exopolysaccharide	Immune modulation
CVF501	C1	Haemophilus iron transport locus	Nutritional/Metabolic factor; Metal uptake; Iron uptake
CVF506	C1	Heme biosynthesis	Nutritional/Metabolic factor
CVF518	C1	Type IV pili biosynthesis	Adherence; Fimbrial adhesin; Type IV pili; Type IVb pili (T4bP)
CVF520	C1	LPS O-antigen (P. aeruginosa)	Immune modulation; Inflammatory signaling pathway
CVF521	C1	Flagella	Motility; Flagella-mediated motility; Flagella assembly
CVF523	C1	Alginate regulation	Biofilm; Biofilm formation
CVF529	C1	GacS/GacA two-component system	Regulation
CVF534	C1	P. syringae TTSS effectors	Effector delivery system
CVF535	C1	Hcp secretion island-1 encoded type VI secretion system (H-T6SS)	Effector delivery system; Type VI secretion system (T6SS); T6SS components
CVF546	C1	Phytotoxin phaseolotoxin	Exotoxin
CVF567	C1	Polysaccharide capsule	Immune modulation; Antiphagocytosis
CVF569	C1	PlcR-PapR quorum sensing	Biofilm
CVF588	C1	PDH-B	Adherence
CVF618	C1	Capsule	Immune modulation; Antiphagocytosis
CVF628	C1	Autoinducer-2	Biofilm; Quorum sensing;
CVF643	C1	Flagella	Motility; Flagella-mediated motility; Flagella assembly
CVF649	C1	MymA operon	Immune modulation

CVF651	C1	Trehalose-recycling ABC transporter	Nutritional/Metabolic factor
CVF655	C1	Zmp1	Exoenzyme
CVF658	C1	Copper exporter	Nutritional/Metabolic factor
CVF660	C1	Nucleoside diphosphate kinase	Immune modulation
CVF667	C1	RegX3	Regulation
CVF736	C1	ACE T6SS	Effector delivery system
CVF757	C1	Phosphoethanolamine modification	Nutritional/Metabolic factor
CVF760	C1	Catalase	Stress survival
CVF775	C1	Capsule	Immune modulation; Antiphagocytosis
CVF779	C1	PbpG	Immune modulation; Complement evasion/Serum resistance
CVF780	C1	Exe T2SS	Effector delivery system; Type II secretion system (T2SS); T2SS components
CVF783	C1	Tap type IV pili	Adherence; Fimbrial adhesin; Type IV pili; Type IVa pili (T4aP)
CVF785	C1	Mannose-sensitive hemagglutinin (Msh) pilus, type IV pili	Adherence; Fimbrial adhesin; Type IV pili; Type IVa pili (T4aP)
CVF786	C1	Polar flagella	Motility; Flagella-mediated motility
CVF792	C1	Hemolysin HlyA	Exotoxin; Membrane-acting toxin; Pore forming toxin (PFT)
CVF793	C1	Hemolysin III	Exotoxin
CVF803	C1	T4SS effectors	Effector delivery system; Type IV secretion system (T4SS); T4SS effectors
CVF827	C1	EF-Tu	Adherence; Non-fimbrial adhesin; Cell wall anchored protein;
CVF833	C1	Capsule	Immune modulation; Antiphagocytosis
CVF834	C1	LPS	Immune modulation; Inflammatory signaling pathway
CVF838	C1	Biotin metabolism	Nutritional/Metabolic factor; Metabolic adaptation; Metabolite
CVF839	C1	Purine	Nutritional/Metabolic factor; Metabolic adaptation; Metabolite

CVF840	C1	Cysteine acquisition	Nutritional/Metabolic factor; Metabolic adaptation; Amino acid uptake
CVF845	C1	Pyrimidine biosynthesis	Nutritional/Metabolic factor; Metabolic adaptation; Metabolite
CVF851	C1	Yersiniabactin	Nutritional/Metabolic factor; Metal uptake; Iron uptake; Siderophore uptake system
CVF852	C1	Aerobactin	Nutritional/Metabolic factor; Metal uptake; Iron uptake; Siderophore uptake system
CVF854	C1	Capsule	Immune modulation; Antiphagocytosis
CVF857	C1	LPS rfb locus	Immune modulation; Inflammatory signaling pathway
CVF859	C1	AcrAB	Antimicrobial activity/Competitive advantage
CVF861	C1	T6SS-II	Effector delivery system
IA009	C1	thioquinolobactin	Siderophore-mediated iron uptake
IA023	C1	vulnibactin	Nutritional/Metabolic factor
IA033	C1	desferrioxamine	Nutritional/Metabolic factor ； Metal uptake； Siderophore-mediated iron uptake
IA044	C1	HasA-type hemophore-mediated heme uptake system	Heme-mediated iron uptake
IA062	C1	Direct heme uptake system	Nutritional/Metabolic factor; Metal uptake; Iron uptake; Heme uptake system;
IA065	C1	Direct heme uptake system	Nutritional/Metabolic factor; Metal uptake; Iron uptake; Heme uptake system;
SS003	C1	T3SS	Effector delivery system
SS016	C1	Cpi-1a + Cpi-1 (SPI-1 like)	Effector delivery system； Type III secretion system (T3SS)
SS025	C1	T3SS	Effector delivery system
SS180	C1	HSI-3	Effector delivery system; Type VI secretion system (T6SS); T6SS components;
SS194	C1	T6SS	Effector delivery system; Type VI secretion system (T6SS); T6SS components
SS208	C1	Yst1 T2SS	Effector delivery system; Type II secretion system (T2SS); T2SS components
SS209	C1	Exe T2SS	Effector delivery system; Type II secretion system (T2SS); T2SS components

SS214	C1	Eps T2SS	Effector delivery system; Type II secretion system (T2SS); T2SS components;
TX171	C1	PlaB	Exotoxin
VF0056	C1	LPS	Immune modulation; Inflammatory signaling pathway
VF0073	C1	ClpE	Stress survival
VF0074	C1	ClpP	Stress survival
VF0082	C1	Type IV pili	Adherence; Fimbrial adhesin; Type IV pili; Type IVa pili (T4aP)
VF0085	C1	LPS	Immune modulation; Inflammatory signaling pathway
VF0103	C1	Agf	Adherence; Fimbrial adhesin; Bacterial amyloid fibers; Curli<br>Biofilm; Biofilm formation
VF0109	C1	SodCI	Stress survival
VF0112	C1	RpoS	Regulation
VF0113	C1	Fur	Regulation
VF0156	C1	Dot/Icm T4SS secreted effectors	Effector delivery system; Type IV secretion system (T4SS); T4SS effectors
VF0167	C1	GspA	Stress survival
VF0168	C1	KatAB	Stress survival
VF0169	C1	SodB	Stress survival
VF0334	C1	HSI-1	Effector delivery system; Type VI secretion system (T6SS); T6SS components
VF0335	C1	T6SS	Effector delivery system; Type VI secretion system (T6SS); T6SS components
VF0361	C1	Capsule	Immune modulation; Antiphagocytosis
VF0368	C1	BvrR-BvrS	Regulation
VF0430	C1	Flagella	Motility; Flagella-mediated motility; Flagella assembly
VF0436	C1	Capsule I	Immune modulation; Antiphagocytosis
VF0463	C1	BfmRS	Regulation
VF0473	C1	Polar flagella	Motility; Flagella-mediated motility
VF0474	C1	Lateral flagella	Motility; Flagella-mediated motility
VF0478	C1	Tap type IV pili	Adherence; Fimbrial adhesin; Type IV pili; Type IVa pili (T4aP)

VF0504	C1	AdeFGH efflux pump	Biofilm formation
VF0519	C1	Flagella	Motility; Flagella-mediated motility
VF0612	C1	Flp pili	Adherence; Fimbrial adhesin; Type IV pili; Type IVc (T4cP) (Tad-type pili)
VF0619	C1	GbpA	Adherence; Non-fimbrial adhesin
CVF258	C2	Type IV pilus	Adherence; Fimbrial adhesin; Type IV pili; Type IVa pili (T4aP)
CVF353	C2	Lvh (Legionella vir homologs) type IVA secretion system	Effector delivery system; Type IV secretion system (T4SS); Type IVA (T4ASS) components
CVF380	C2	LPS	Immune modulation; Inflammatory signaling pathway
CVF551	C2	Pyoverdine	Nutritional/Metabolic factor; Metal uptake; Iron uptake; Siderophore uptake system
CVF553	C2	Pyochelin	Nutritional/Metabolic factor; Metal uptake; Iron uptake; Siderophore uptake system
CVF604	C2	Trw T4SS	Effector delivery system; Type IV secretion system (T4SS); Type IVA (T4ASS) components
CVF607	C2	Virulence-associated proteins	Others
CVF737	C2	ABC transporter for dispersin	Others
CVF891	C2	B. cereus exo-polysaccharide (BPS)	Immune modulation
IA010	C2	Alcaligin	Siderophore-mediated iron uptake
IA059	C2	Direct heme uptake system	Nutritional/Metabolic factor; Metal uptake; Iron uptake; Heme uptake system;
SS015	C2	T3SS	Effector delivery system
SS026	C2	T3SS	Effector delivery system
SS051	C2	CoxH2/rimL	Effector delivery system; Type IV secretion system (T4SS); T4SS effectors
VF0079	C2	Capsule	Immune modulation; Antiphagocytosis
VF0228	C2	Enterobactin	Nutritional/Metabolic factor; Metal uptake; Iron uptake; Siderophore uptake system

VF0372	C2	Trw T4SS	Effector delivery system; Type IV secretion system (T4SS); Type IVA (T4ASS) components
VF0432	C2	CdpA	Regulation
VF0475	C2	Tap type IV pili	Adherence; Fimbrial adhesin; Type IV pili; Type IVa pili (T4aP)
VF0515	C2	MSHA pili	Adherence; Fimbrial adhesin; Type IV pili; Type IVa pili (T4aP)
VF0542	C2	LPS	Immune modulation; Inflammatory signaling pathway
VF0573	C2	Colibactin	Exotoxin; Intracellularly active toxin; DNase/Genotoxin
VF0615	C2	VPS	Biofilm; Biofilm formation
AI059	C3	Stb	Adherence
AI085	C3	CupA fimbriae	Adherence; Fimbrial adhesin;
AI088	C3	CupD fimbriae	Adherence; Fimbrial adhesin;
VF0336	C3	BvgAS	Regulation
CVF849	C4	Ent siderophore	Nutritional/Metabolic factor; Metal uptake; Iron uptake; Siderophore uptake system
SS182	C4	AAI/SCI-II T6SS	Effector delivery system
VF0477	C4	MSHA type IV pili	Adherence; Fimbrial adhesin; Type IV pili; Type IVa pili (T4aP)

Table S4. Predicted plasmid replicons distributed in *S. algae*

**Strain**	**Sequence ID**	**RefSeq ID**	**Identity (%)**	**Coverage length (bp)**	**Plasmid size (bp)**	**plasmid replicon (accession No.)**	**Antimicrobial resistance genes**	**Virulence-associated genes**
13	Scaffold20	NZ_CP014052.1	97.01	12,089	217,123	—	*aadA3*, *bla*$_{OXA-4}$, *aac(6')-Ib4*, *dfrA1*, *qacL*, *aph(6)-Id*, *tet(A)*	*tppE*

14	Scaffold17	NZ_CP100424.1	99.99	79,378	198,507	IncC_1, JN157804	*qacEdelta1*, *aac(3)-IIa*, *sul2*	*cdpA*
14	Scaffold26	NZ_CP100424.1	100	23,168	198,507	IncC_1, JN157804	—	*trwD*, *trwE*
6	Scaffold24	NZ_OQ622009.1	99.97	11,929	308,785	—	$bla_{TEM-210}$, *ant(3'')-IIa*, *sul1*, *qacEdelta1*	*lpxA/glmU*
A41	JADZHC010000098.1	NZ_CP014052.1	97.29	10,047	217,123	—	—	—
A41	JADZHC010000099.1	CP055413.1	96.67	10,486	481,290	IncFIB(K)_1, JN233704	—	*vapA3*
ATCC51192	JAAXPX010000027.1	NZ_CP090942.1	98.49	26,111	64,388	—	—	—
CCU101	CP018457.1	NZ_CP032414.1	99.99	75,733	132,551	IncC_1, JN157804	$bla_{TEM-210}$, *qacEdelta1*, *sul1*, *floR*, *NmcR*, *tet(A)*, *aph(6)-Id*, *aph(3'')-Ib*,*sul2*	—
CCU4051	CP078514.1	NZ_CP032414.1	99.99	75,733	132,551	IncC_1, JN157804	$bla_{TEM-210}$, *qacEdelta1*, *sul1*, *floR*, *NmcR*, *tet(A)*, *aph(6)-Id*, *aph(3'')-Ib*, *sul2*	—

CCU4052	CP078516.1	NZ_CP032414.1	99.99	95,361	132,551	IncC_1, JN157804	*bla*$_{TEM-210}$, *qacEdelta1*, *sul1*, *floR*, *NmcR*, *tet(A)*, *aph(6)-Id*, *aph(3'')-Ib*, *sul2*	—
CCU4053	CP078517.1	NZ_CP032414.1	100	132,551	132,551	IncC_1, JN157804	*NmcR*, *floR*, *sul1*, *qacEdelta1*, *bla*$_{TEM-210}$, *sul2*, *aph(3'')-Ib*, *aph(6)-Id*	—
CCU4054	CP078520.1	NZ_CP032414.1	99.99	93,136	132,551	IncC_1, JN157804	*bla*$_{TEM-210}$, *qacEdelta1*, *sul1*, *floR*, *NmcR*, *tet(A)*, *aph(6)-Id*, *aph(3'')-Ib*, *sul2*	—
CHL	LVDF01000010.1	NZ_CP014052.1	97.24	12,051	217,123	—	—	*carA*, *carB*, *CBU_1434*, *dnaK*, *mip*, *mrsA/glmM*, *tppE*
CLS1	LTBI01000025.1	NZ_CP043190.1	99.99	52,153	165,649	IncC_1, JN157804	*erm(42)*, *bla*$_{TEM-2}$, *aph(6)-Id*, *aph(3'')-Ib*, *sul2*, *bla*$_{CMY-2}$	*PA3142*
CLS1	LTBI01000028.1	NZ_CP103514.1	99.93	52,114	190,614	IncC_1, JN157804	—	*trwD*, *trwE*
CLS1	LTBI01000074.1	NZ_CP048305.1	100	18,318	159,959	IncC_1, JN157804	—	—
CSBBB	CP047421.1	NZ_CP031610.1	99.84	64,479	160,292	IncC_1, JN157804	*bla*$_{TEM-102}$, *dfrA12*, *qacEdelta1*, *sul1*, *bla*$_{CTX-M-}$	—

							$_{84}$, *armA*, $bla_{TEM-210}$, $bla_{CTX-M-103}$	
DC18SW04	JAVMLJ010000001.1	NZ_CP087332.1	99.98	12,171	148,957	—	*golS, soxR, MexH, MexI, aph(6)-Id, ugd, TxR, tet(35), H-NS, adeH*	*A225_RS19005, cheA-2, cheB-2, cheR-3, cheV, cheW-2, cheY, cheZ, ctpV, flaA, flaB, fleI/flaG, fleR/flrC, fleS/flrB, flgA, flgB, flgD, flgE, flgF, flgG, flgH, flgI, flgJ, flgM, flgN, flgP, flhA, flhB, flhF, fliA, fliE, fliG, fliH, fliI, fliJ, fliL, fliM, fliO, fliP, fliQ, fliR, fliS, flrA, galU, gndA, hcp, hemH, hemN, icl, katA, LPG_RS13035, maf-2, manB/yhxB, orfM, pseB, pseC, rmlA, rmlB, rmlC, rmlC, rmlD, STU_RS14610, tapU, ugd, vgrG-2, wecA*
DC19SW04	JAVMKZ010000040.1	CP055413.1	96.86	10,486	481,290	IncFIB(K)_1, JN233704	—	—
DC19SW04	JAVMKZ010000049.1	NZ_CP014052.1	96.74	12,117	217,123	—	—	—
HUDG3	JADZGS010000025.1	NZ_CP014052.1	96.64	12,122	217,123	—	*floR, adeH, H-NS, tet(35)*	*hemH, hemN, orfM, papR, STU_RS14610, tapU, tppE, vapA3*
JC874	JAWJDV010000011.1	NZ_CP051530.1	93.77	11,518	194,145	—	*cprR*	*ctpV, epsD, epsE, ETAE_RS04220, exeC, exeF, exeG, exeJ, exeK, exeL, VV1_RS15610, yst1I*

JCM14758	BALL01000004.1	NZ_MH457126.1	92.02	27,844	167,140	IncC_1, JN157804	*golS, mdsA, adeF, mdsC, adeR, TriC*	*adeF, adeG, ctpV, lpxC*
JFC1	LUJI01000012.1	NZ_CP014052.1	96.81	11,299	217,123	—	—	*tppE*
KCNaR1	CP033574.1	NZ_MF627445.1	99.93	12,268	192,736	IncC_1, JN157804	*aac(6')-Ie-aph(2'')-Ia, tet(A), aph(6)-Id, arr-2, dfrA27, aadA16, qacEdelta1, sul1, ant(3'')-IIa, $bla_{TEM-210}$, qnrA1, aadA, bla_{OXA-10}, cmlA5, ant(2'')-Ia, veb-1*	*cdpA*
LC201651	DAOKES010000045.1	NZ_MW574944.1	99.65	16,630	50,478	IncU_1, DQ401103	—	*trwK, virB10, virB11, virD4*
LC201652	JAGQAO010000030.1	NZ_MW574944.1	99.63	16,630	50,478	IncU_1, DQ401103	—	*trwK, virB10, virB11, virD4*
LC20166	JAGQAN010000062.1	NZ_MW574944.1	99.66	16,566	50,478	IncU_1, DQ401103	—	*trwK, virB10, virB11, virD4*

NCTC10738	UGYO01000001.1	NZ_CP014052.1	97.32	10,576	217,123	—	*bla*$_{\text{TEM-208}}$, *TxR*, *tet(35)*, *bla*$_{\text{CTX-M-84}}$, *H-NS*, *adeH*, *floR*, *aph(6)-Id*, *CRP*, *TolC*, *ParR*, *aac(6')-Ib7*, *adeF*, *cprR*, *acrD*, *MexD*, *aac(3)-IIb*, *qnrA3*, *bla*$_{\text{OXA-405}}$, *adeL*, *FosA*	*acrB*, *AHA_RS09305*, *algU*, *algW*, *ASA_RS16580*, *BJE04_RS21750*, *carA*, *carB*, *CBU_1434*, *CBU_1594*, *ccmB*, *ccmC*, *ccmE*, *ccmF*, *cheA*, *cheA-2* *cheB*, *cheB-2*, *cheD*, *cheR*, *cheR-3*, *cheV*, *cheW*, *cheW-2*, *cheY*, *cheY1*, *cheZ*, *clpB/vasG*, *csgG*, *cysC1*, *dnaK*, *DNO_RS02105*, *epsD*, *epsE*, *ETAE_RS04220*, *exeA*, *acrA*, *exeC*, *exeF*, *exeG*, *exeJ*, *exeK*, *exeL*, *fleI/flaG*, *fleR*, *fleR/flrC*, *fleS/flrB*, *flgA*, *flgB*, *flgC*, *flgD*, *flgE*, *flgF*, *flgFL*, *flgG*, *flgH*, *flgI*, *flgJ*, *flgM*, *flgML*, *flgN*, *flgNL*, *flgP*, *flhA*, *flhB*, *flhF*, *fliA*, *fliC*, *fliE*, *fliEL*, *fliF*, *fliG*, *fliGL*, *fliH*, *fliI*, *fliIL*, *fliJ*, *fliL*, *fliM*, *fliNL*, *fliO*, *fliP*, *fliPL*, *fliQ*, *fliR*, *fliS*, *flmH*, *flrA*, *galU*, *gbpA*, *glnA1*, *gmhA*, *gndA*, *hcp-2*, *hemA*, *hemB*, *hemC*, *hemE*, *hemG*, *hemH*, *hemL*, *hemN*, *hitA*, *hitB*, *hitC*, *hlyA*, *htpB*, *htrB*, *hutX*, *hutZ*, *huvC*, *icmF/vasK*, *irgA*, *JJD26997_RS08230*, *katA*, *katB*, *kdkA*, *kdsA*, *kdtB*, *lafA*, *lafC*, *lafS*, *lafT*, *leuD*, *lfgG*, *lfhA*, *lfiR*, *LPC_RS08370*, *LPG_RS13035*, *LPG_RS14840*, *lpxA/glmU*, *lpxC*, *luxS*, *MGA_RS01700*, *mip*, *motX*, *mprA*, *mrsA/glmM*, *mshA*, *mshD*, *mshE*, *mshG*, *mshJ*, *mshL*, *mshM*, *mshN*, *mshQ*,

								opsX/rfaC, orfM, PA14_RS29920, panC, papR, pbpG, pchH, phoP, pilA, pilM, pilN, pilO, pilR, plr/gapA, prrA, pyrB, qbsC, rfaE, rmlA, rmlB, rpe, rpoN, sodCI, STU_RS14610, sugC, tagT, tapB, tapC, tapD/pilD, tapQ, tapU, tolC, tppE, tufA, vapA3, vasA, vasB, vasD, vasE, vasF, vasH, vasI, vasJ, VCA0109, vfr, vgrG-1, vgrG-2, vipA/mglA, vipB/mglB, VP_RS16580, VP_RS22390, VP_RS22495, VP_RS22515, VP_RS22610, VV1_RS15610, waaA, wbfU, wbfY, ybtP, yst1I, zmp1
S12	Scaffold22	CP104923.1	100	64,938	157,034	IncC_1, JN157804	*sul2, aph(3'')-Ib, aph(6)-Id*	—
S12	Scaffold23	NZ_CP096167.1	99.99	42,178	137,224	IncC_1, JN157804	—	—
S12	Scaffold29	NZ_OQ230783.1	100	13,379	20,817	—	*qacEdelta1, sul1, BRP(MBL), NDM-1, adeN, catB3, ant(3'')-IIa,* $bla_{TEM-210}$*, mphA*	*SAUSA300_RS00840*
S150735	CP068229.1	NZ_CP014052.1	97.23	11,896	217,123	—	*TxR, adeF, acrA, aac(6')-Ib7, cprR, parR, farA, tet(35), acrD, MexD, aac(3)-IIb, qnrA3, macB,* $bla_{OXA-405}$*, adeL, FosA,*	*A225_RS19005, ABSDF_RS00350, ABTW07_0084, ABZJ_RS06230, acpXL, acrA, acrB, adeG, adhD, AHA_RS09305, AHML_RS18555, ahpC, algU, algW, ASA_RS16580, bioB, BJAB07104_RS00525,*

		DfrA42, ParS, tet(C), kdpE, baeR, rsmA, msbA, YajC, evgS, EdeQ, KpnF, KpnE, mtrC, macA, hmrM, MCR-4.3, emrE, aac(6')-Ip, golS, soxR, MexH, MexI, ugd, H-NS, adeH, cmlA9, sul2, aph(3'')-Ib, aph(6)-Id, NmcR, floR, sul1, qacEdelta1, tet(59), fox-7, catB9, CRP, TolC, rpoB	*BJE04_RS21750, bvgA, bvrR, carA, carB, CBU_1434, CBU_1566, CBU_1594, CBUG_RS08820, ccmB, ccmC, ccmE, ccmF, cheA, cheA-2, cheB, cheB-2, cheD, cheR, cheR-3, cheV, cheW, cheW-2, cheY, cheY1, cheZ, clpB/vasG, clpE, clpP, cpsA/uppS, cpsB/cdsA, csgG, ctpV, cupA2, cupA3, CV_RS12900, cysC1, dnaK, DNO_RS02105, EAMY_RS32245, EAMY_RS32250, EAMY_RS32255, eno, epsD, epsE, ETAE_RS04220, exeA, exeC, exeF, exeG, exeJ, exeK, exeL, fabZ, fauA, feoB, fepD, fepG, flaA, flaB, fleI/flaG, fleQ, fleR, fleR/flrC, fleS/flrB, flgA, flgB, flgC, flgD, flgE, flgF, flgFL, flgG, flgH, flgI, flgJ, flgM, flgML, flgN, flgP, flhA, flhB, flhF, fliA, fliE, fliEL, fliF, fliG, fliGL, fliH, fliI, fliIL, fliJ, fliL, fliM, fliNL, fliO, fliP, fliPL, fliQ, fliR, fliS, flmH, flrA, fur, gacA, galE, galU, gbpA, ggt, glnA1, gmhA, gndA, hcp, hcp-2, hemA, hemB, hemC, hemE, hemG, hemH, hemL, hemN, hitA, hitB, hitC, hlyA, hopAJ2, htpB, htrB, hutX, hutZ, huvC, icl, icmF/vasK, irgA, iutA, JJD26997_RS08230, kasB, katA, katB, kdkA, kdsA, kdsB, kdtB, lafA, lafC, lafK, lafS, lafT, lap, leuD, lfgG,*

								lfhA, lfiR, LPC_RS08370, LPG_RS11000, LPG_RS13035, LPG_RS14840, lptA, lpxA, lpxA/glmU, lpxB, lpxC, lpxD, lpxH, lpxK, luxS, maf-2, manB, manB/yhxB, MGA_RS01700, mip, motA, motX, motY, mprA, mrsA/glmM, msbA, mshA, mshB, mshD, mshE, mshG, mshL, mshM, mshQ, mucP, ndk, neuB2, neuC, opsX/rfaC, orfM, PA14_RS24375, PA2359, PA3349, panC, papR, pbpG, PD_RS07885, pdhB, pgi, phoP, pilE3, pilM, pilN, pilO, pilP, pilR, plr/gapA, PM_RS08640, prrA, pseB, pseC, PSPA7_RS30430, PSPTO_RS07240, purM, pyrB, qbsC, regX3, relA, rfaE, rmlA, rmlB, rmlC, rpe, rpoN, rpoS, sodB, sodCI, stbA, STU_RS14610, sugC, tagT, tapB, tapC, tapD/pilD, tapQ, tapU, tolC, tppE, tufA, ugd, vasA, vasB, vasD, vasE, vasF, vasH, vasI, vasJ, VCA0109, vfr, vgrG-1, vgrG-2, vipA/mglA, vipB/mglB, viuC, VP_RS16580, VP_RS22390, VP_RS22495, VP_RS22515, VP_RS22610, VV1_RS15610, waaA, wcbN, wecA, ybtP, YE105_RS07900, yst1I, zmp1
S33	Scaffold12	NZ_CP014052.1	97.09	12,050	217,123	—	—	*carA, carB, CBU_1434, dnaK, mip, mrsA/glmM, papR, tppE*

S35	Scaffold14	NZ_LT985220.1	97.37	29,673	174,073	IncC_1, JN157804	—	—
S404	JADZHZ010000055.1	NZ_CP014052.1	97.3	11,402	217,123	—	—	—
S7	Scaffold14	NZ_CP096167.1	99.99	119,155	137,224	IncC_1, JN157804	*sul2, aph(3'')-Ib, aph(6)-Id*	—
S97087	JADZHJ010000023.1	NZ_CP051530.1	93.76	11,518	194,145	—	*bla*$_{CTX\text{-}M\text{-}84}$	*ctpV*
Sh392	QFDC01000013.1	NZ_CP014052.1	97.48	11,301	217,123	—	—	*tppE*
VGH1171	CP032414.1	NZ_CP032414.1	100	132,551	132,551	IncC_1, JN157804	*NmcR, floR, sul1, qacEdelta1, bla*$_{TEM\text{-}210}$*, sul2, aph(3'')-Ib, aph(6)-Id*	—

Table S5. The results of integrons identified in *S. algae* genomes

NO.	Strain	Contig ID	Integron type	Number of attC elements	Number of protein
1	5	Scaffold14	Complete	2	4
2	6	Scaffold24	Complete	1	3
3	6	Scaffold4	In0	0	1
4	6	Scaffold47	CALIN	2	2
5	12	Scaffold14	Complete	2	4
6	13	Scaffold12	CALIN	4	6
7	13	Scaffold20	Complete	2	6
8	13	Scaffold4	CALIN	2	3
9	14	Scaffold19	Complete	2	6
10	14	Scaffold2	CALIN	2	2
11	6638	Scaffold1	In0	0	1
12	6638	Scaffold1	CALIN	2	3
13	6638	Scaffold5	CALIN	2	2
14	2NE11	CP055159.1	In0	0	1
15	A292	JADZHD010000022.1	Complete	1	4
16	A292	JADZHD010000030.1	CALIN	3	7
17	A292	JADZHD010000031.1	CALIN	2	3
18	A41	JADZHC010000065.1	CALIN	2	0
19	A56	JADZHB010000016.1	CALIN	2	2
20	A56	JADZHB010000024.1	Complete	1	3
21	A56	JADZHB010000024.1	CALIN	3	3
22	A56	JADZHB010000028.1	CALIN	2	2
23	A57	JADZHA010000022.1	CALIN	3	6
24	A58	JADZGZ010000046.1	In0	0	1
25	A58	JADZGZ010000065.1	CALIN	2	2
26	A58	JADZGZ010000088.1	CALIN	2	2
27	A59	CP068227.1	Complete	1	3
28	A59	CP068227.1	CALIN	2	3
29	A65	JADZGX010000036.1	CALIN	2	3
30	A93	JADZGW010000016.1	CALIN	2	2
31	A93	JADZGW010000019.1	Complete	2	3
32	A93	JADZGW010000020.1	CALIN	3	5
33	A94	JADZGV010000019.1	CALIN	2	2
34	A94	JADZGV010000020.1	Complete	3	4
35	A94	JADZGV010000023.1	CALIN	2	2
36	A97	JADZGU010000026.1	CALIN	2	2

37	A97	JADZGU010000034.1	In0	0	1
38	AC	LVDH01000001.1	In0	0	1
39	AC	LVDH01000007.1	CALIN	2	2
40	ATCC49138	AP024609.1	Complete	1	3
41	ATCC49138	AP024609.1	CALIN	2	2
42	ATCC49138	AP024609.1	In0	0	1
43	ATCC51192	JAAXPX010000001.1	Complete	5	9
44	B2215466	CP110338.1	In0	0	1
45	B29	JAMQCQ010000041.1	CALIN	2	3
46	BrY	MDKA01000103.1	CALIN	3	4
47	C6G3	JPMA01000020.1	CALIN	2	3
48	CCU101	CP018456.1	In0	0	1
49	CCU4051	CP078513.1	In0	0	1
50	CCU4052	CP078515.1	In0	0	1
51	CCU4053	CP078518.1	In0	0	1
52	CCU4054	CP078519.1	In0	0	1
53	CCUG12945	JADZHV010000015.1	CALIN	2	3
54	CCUG12945	JADZHV010000016.1	In0	0	1
55	CCUG12945	JADZHV010000021.1	CALIN	2	3
56	CCUG12945	JADZHV010000022.1	CALIN	3	4
57	CCUG15259	JADZHU010000020.1	In0	0	1
58	CCUG20533	JADZHT010000030.1	In0	0	1
59	CCUG38646	JADZHQ010000012.1	Complete	2	5
60	CCUG38646	JADZHQ010000015.1	CALIN	3	4
61	CCUG38646	JADZHQ010000020.1	CALIN	3	4
62	CCUG38646	JADZHQ010000020.1	CALIN	3	7
63	CCUG48086	JADZHP010000015.1	In0	0	1
64	CCUG50501	JADZHO010000018.1	Complete	1	3
65	CCUG526	JADZHY010000017.1	Complete	1	3
66	CCUG56496	JADZHN010000017.1	In0	0	1
67	CCUG56496	JADZHN010000022.1	CALIN	2	3
68	CCUG58400	JADZHM010000030.1	CALIN	2	3
69	CCUG72638	JADZHL010000015.1	CALIN	2	7
70	CCUG72638	JADZHL010000027.1	CALIN	2	3
71	CCUG72638	JADZHL010000027.1	CALIN	2	3
72	CCUG72638	JADZHL010000029.1	CALIN	2	1
73	CCUG72638	JADZHL010000040.1	CALIN	2	3
74	CCUG72678	JADZHK010000025.1	CALIN	2	3
75	CCUG789	JADZHX010000026.1	Complete	3	7
76	CCUG789	JADZHX010000035.1	CALIN	3	4

77	CECT5071	CP068230.1	Complete	5	9
78	CHL	LVDF01000033.1	Complete	2	5
79	CLS1	LTBI01000004.1	In0	0	1
80	CLS1	LTBI01000032.1	CALIN	3	3
81	CLS1	LTBI01000080.1	Complete	3	4
82	CLS1	LTBI01000127.1	CALIN	2	3
83	CLS1	LTBI01000136.1	CALIN	2	3
84	CLS2	LVDV01000005.1	CALIN	2	4
85	CLS2	LVDV01000008.1	CALIN	2	3
86	CLS2	LVDV01000056.1	In0	0	1
87	CLS3	LVDX01000030.1	In0	0	1
88	CSB04KR	MBFW01000008.1	CALIN	2	6
89	CSB04KR	MBFW01000036.1	In0	0	1
90	CSBBB	CP047421.1	Complete	2	3
91	DC17SW01	JAVMLV010000008.1	In0	0	1
92	DC17SW01	JAVMLV010000016.1	CALIN	3	7
93	DC17SW01	JAVMLV010000037.1	Complete	1	3
94	DC17SW02	JAVMLU010000037.1	CALIN	2	2
95	DC17SW02	JAVMLU010000051.1	In0	0	1
96	DC17SW03	JAVMLT010000036.1	CALIN	2	2
97	DC17SW03	JAVMLT010000053.1	In0	0	1
98	DC17SW04	JAVMLS010000037.1	CALIN	2	2
99	DC17SW04	JAVMLS010000056.1	In0	0	1
100	DC17SW05	JAVMLR010000086.1	CALIN	2	3
101	DC17SW06	JAVMLQ010000088.1	CALIN	2	3
102	DC17SW07	JAVMLP010000041.1	Complete	4	6
103	DC17SW08	JAVMLO010000042.1	CALIN	2	2
104	DC18SW01	JAVMLM010000080.1	Complete	2	6
105	DC18SW03	JAVMLK010000029.1	CALIN	2	2
106	DC18SW03	JAVMLK010000032.1	CALIN	2	2
107	DC18SW03	JAVMLK010000039.1	In0	0	1
108	DC18SW04	JAVMLJ010000043.1	In0	0	1
109	DC18SW07	JAVMLG010000010.1	In0	0	1
110	DC18SW07	JAVMLG010000031.1	CALIN	2	3
111	DC18SW07	JAVMLG010000044.1	CALIN	2	3
112	DC18SW09	JAVMLE010000015.1	CALIN	4	7
113	DC18SW09	JAVMLE010000098.1	CALIN	2	3
114	DC18SW10	JAVMLD010000010.1	In0	0	1
115	DC18SW10	JAVMLD010000026.1	CALIN	2	3
116	DC18SW10	JAVMLD010000070.1	CALIN	2	3

117	DC19SW01	JAVMLC010000009.1	In0	0	1
118	DC19SW01	JAVMLC010000024.1	CALIN	2	3
119	DC19SW04	JAVMKZ010000005.1	CALIN	2	3
120	DC19SW04	JAVMKZ010000063.1	Complete	3	5
121	DC19SW05	JAVMKY010000017.1	Complete	2	5
122	DC19SW05	JAVMKY010000026.1	CALIN	2	4
123	DC19SW05	JAVMKY010000032.1	CALIN	3	4
124	DC19SW05	JAVMKY010000037.1	CALIN	2	3
125	DC19SW05	JAVMKY010000058.1	CALIN	2	2
126	DC19SW06	JAVMKX010000019.1	Complete	2	5
127	DC19SW06	JAVMKX010000024.1	CALIN	2	4
128	DC19SW06	JAVMKX010000028.1	CALIN	2	3
129	DC19SW06	JAVMKX010000034.1	CALIN	3	4
130	DC19SW06	JAVMKX010000056.1	CALIN	2	2
131	EBI	OY771610.1	CALIN	2	4
132	G1	CP068228.1	In0	0	1
133	HT103	Scaffold22	Complete	1	3
134	HUDH4	JADZGR010000026.1	In0	0	1
135	HUDI2	JADZGQ010000024.1	In0	0	1
136	INSAq258	JAKCOE010000030.1	In0	0	1
137	INSAq494	JAKCOU010000022.1	CALIN	2	1
138	INSAq494	JAKCOU010000039.1	CALIN	2	3
139	INSAq494	JAKCOU010000085.1	In0	0	1
140	INSAq494	JAKCOU010000095.1	CALIN	3	7
141	INSAq495	JAKCOV010000074.1	In0	0	1
142	JC874	JAWJDV010000001.1	In0	0	1
143	JCM14758	BALL01000035.1	CALIN	2	4
144	JCM14758	BALL01000080.1	In0	0	1
145	JCM21037	BALO01000058.1	Complete	4	6
146	JFC2	LUKM01000014.1	CALIN	2	2
147	JFC2	LUKM01000019.1	CALIN	2	2
148	JFC2	LUKM01000031.1	In0	0	1
149	JFC3	LVCX01000017.1	CALIN	2	3
150	JFL	LVDI01000009.1	CALIN	3	5
151	JFL	LVDI01000019.1	CALIN	2	2
152	KCNaR1	CP033574.1	Complete	2	5
153	KCNaR1	CP033574.1	Complete	4	9
154	KCNaR1	CP033575.1	CALIN	2	3
155	LC20161	JAGQAS010000033.1	In0	0	1
156	LC20161	JAGQAS010000102.1	CALIN	3	4

157	LC20161	JAGQAS010000111.1	CALIN	2	5
158	LC20162	JAGQAR010000053.1	CALIN	3	4
159	LC20162	JAGQAR010000100.1	In0	0	1
160	LC20163	JAGQAQ010000023.1	CALIN	2	5
161	LC20163	JAGQAQ010000054.1	CALIN	3	4
162	LC20163	JAGQAQ010000102.1	In0	0	1
163	LC20164	JAGQAP010000030.1	In0	0	1
164	LC20164	JAGQAP010000105.1	CALIN	3	4
165	LC201651	DAOKES010000002.1	CALIN	3	8
166	LC201651	DAOKES010000006.1	In0	0	1
167	LC201651	DAOKES010000068.1	CALIN	2	3
168	LC201652	JAGQAO010000006.1	CALIN	3	7
169	LC201652	JAGQAO010000035.1	CALIN	2	3
170	LC201652	JAGQAO010000073.1	In0	0	1
171	LC20166	JAGQAN010000008.1	In0	0	1
172	LC20166	JAGQAN010000015.1	CALIN	3	7
173	LC20166	JAGQAN010000063.1	CALIN	2	3
174	LC4	JASSPJ010000004.1	CALIN	4	5
175	LCUVS1	JAUTEG010000002.1	CALIN	2	5
176	LZ2013652	JAGQAL010000010.1	In0	0	1
177	LZ2013652	JAGQAL010000050.1	CALIN	2	3
178	LZ2015243	JAGQAK010000013.1	CALIN	2	5
179	LZ2015243	JAGQAK010000030.1	CALIN	2	3
180	LZ2015256	JAGQAJ010000016.1	Complete	1	3
181	LZ2015256	JAGQAJ010000053.1	CALIN	2	4
182	LZ2015256	JAGQAJ010000070.1	CALIN	2	2
183	LZ2015256	JAGQAJ010000137.1	CALIN	2	2
184	MARS	LN810019.1	In0	0	1
185	MARS	LN810019.1	CALIN	2	2
186	MAS2314	DAOKEQ010000028.1	CALIN	2	2
187	MAS23142	JAGQAT010000023.1	CALIN	2	2
188	MAS2736	DAOKET010000083.1	In0	0	1
189	MAS2736	DAOKET010000113.1	CALIN	2	6
190	MAS27361	JAGQAI010000002.1	CALIN	2	6
191	MAS27361	JAGQAI010000006.1	In0	0	1
192	MCCC1A11468	CP129401.1	In0	0	1
193	melkephy	LVDG01000001.1	In0	0	1
194	melkephy	LVDG01000041.1	CALIN	2	3
195	MN01	LIRM01000048.1	In0	0	1
196	NBRC1031	BCZT01000080.1	Complete	5	9

197	OTH19	ABLONE010000001.1	CALIN	2	2
198	OTH19	ABLONE010000024.1	Complete	1	3
199	OTH19	ABLONE010000024.1	CALIN	3	3
200	OTH19	ABLONE010000039.1	CALIN	2	2
201	RC	LVCZ01000020.1	CALIN	3	3
202	RC	LVCZ01000026.1	CALIN	2	2
203	S1	Scaffold7	In0	0	1
204	S10	Scaffold11	CALIN	2	5
205	S10	Scaffold21	CALIN	2	3
206	S11	Scaffold11	CALIN	2	5
207	S11	Scaffold20	CALIN	2	3
208	S12	Scaffold16	In0	0	1
209	S12	Scaffold34	Complete	1	2
210	S13	Scaffold8	CALIN	3	4
211	S14	Scaffold31	Complete	1	4
212	S15	Scaffold3	CALIN	2	4
213	S15	Scaffold8	In0	0	1
214	S150735	CP068229.1	Complete	1	3
215	S159418	JADZHI010000032.1	CALIN	2	4
216	S159418	JADZHI010000032.1	CALIN	2	3
217	S16	Scaffold18	In0	0	1
218	S17	Scaffold8	CALIN	3	4
219	S19	Scaffold40	Complete	2	6
220	S2	Scaffold22	Complete	1	4
221	S2	Scaffold31	CALIN	2	5
222	S2	Scaffold31	CALIN	3	2
223	S20	Scaffold20	CALIN	2	2
224	S20	Scaffold34	Complete	1	2
225	S2023R	NIJL01000046.1	CALIN	2	3
226	S21	Scaffold39	CALIN	2	3
227	S24	Scaffold13	CALIN	2	2
228	S24	Scaffold14	CALIN	2	2
229	S24	Scaffold21	CALIN	2	4
230	S24	Scaffold24	CALIN	2	4
231	S2541	JAKILW010000010.1	CALIN	2	3
232	S2541	JAKILW010000010.1	CALIN	2	4
233	S26	Scaffold13	CALIN	2	2
234	S26	Scaffold41	CALIN	3	5
235	S26	Scaffold48	Complete	2	6
236	S27	Scaffold21	CALIN	2	6

237	S27	Scaffold3	CALIN	3	7
238	S28	Scaffold3	CALIN	6	5
239	S28	Scaffold6	In0	0	1
240	S28011	JADZHR010000017.1	CALIN	2	2
241	S28011	JADZHR010000027.1	CALIN	2	3
242	S28011	JADZHR010000028.1	CALIN	3	5
243	S3	Scaffold23	Complete	1	4
244	S3	Scaffold33	CALIN	3	2
245	S3	Scaffold33	CALIN	2	5
246	S31	Scaffold12	CALIN	2	4
247	S31	Scaffold14	CALIN	2	3
248	S31	Scaffold14	In0	0	1
249	S31	Scaffold32	CALIN	2	3
250	S32	Scaffold27	In0	0	1
251	S32	Scaffold32	CALIN	2	3
252	S34	Scaffold1	In0	0	1
253	S34	Scaffold2	CALIN	2	2
254	S35	Scaffold10	CALIN	2	3
255	S36	Scaffold3	CALIN	2	4
256	S36	Scaffold4	CALIN	2	3
257	S37	Scaffold8	In0	0	1
258	S4	Scaffold23	Complete	1	4
259	S4	Scaffold32	CALIN	2	5
260	S4	Scaffold32	CALIN	3	2
261	S40	Scaffold25	CALIN	2	3
262	S404	JADZHZ010000032.1	Complete	1	3
263	S404	JADZHZ010000038.1	CALIN	2	5
264	S41	Scaffold1	CALIN	2	2
265	S41	Scaffold29	CALIN	3	3
266	S41	Scaffold33	Complete	1	5
267	S41	Scaffold9	CALIN	2	4
268	S42	Scaffold25	CALIN	2	2
269	S42	Scaffold7	Complete	1	4
270	S43	Scaffold2	CALIN	2	2
271	S43	Scaffold3	In0	0	1
272	S5	Scaffold24	Complete	1	4
273	S5	Scaffold34	CALIN	2	5
274	S5	Scaffold34	CALIN	3	2
275	S5043	JADZHW010000023.1	In0	0	1
276	S5043	JADZHW010000040.1	CALIN	2	2

277	S590722	JADZHH010000027.1	In0	0	1
278	S590722	JADZHH010000040.1	CALIN	2	1
279	S6	Scaffold32	CALIN	2	3
280	S669801	JADZHG010000016.1	CALIN	2	3
281	S7	Scaffold15	In0	0	1
282	S7	Scaffold15	CALIN	2	4
283	S7	Scaffold26	Complete	1	3
284	S7	Scaffold5	CALIN	2	2
285	S8	Scaffold25	CALIN	2	1
286	S8	Scaffold25	In0	0	1
287	S9	Scaffold11	CALIN	2	5
288	S9	Scaffold21	CALIN	2	3
289	S950570	JADZHF010000022.1	CALIN	2	2
290	S950570	JADZHF010000022.1	CALIN	2	3
291	S97087	JADZHJ010000048.1	CALIN	2	2
292	S97087	JADZHJ010000053.1	CALIN	3	7
293	S97087	JADZHJ010000073.1	CALIN	3	2
294	SF7	JADZGP010000016.1	Complete	1	4
295	SF7	JADZGP010000025.1	CALIN	2	5
296	Sh392	QFDC01000015.1	CALIN	3	7
297	SY1	Scaffold44	CALIN	2	3
298	SY101	Scaffold46	CALIN	2	2
299	SY101	Scaffold48	CALIN	2	2
300	SY102	Scaffold14	CALIN	3	4
301	SY102	Scaffold26	In0	0	1
302	SY102	Scaffold31	CALIN	2	4
303	SY103	Scaffold23	CALIN	2	5
304	SY103	Scaffold27	In0	0	1
305	SY104	Scaffold2	CALIN	2	2
306	SY104	Scaffold4	In0	0	1
307	SY11	Scaffold20	CALIN	3	5
308	SY11	Scaffold30	In0	0	1
309	SY15	Scaffold22	CALIN	2	5
310	SY15	Scaffold27	In0	0	1
311	SY17	Scaffold15	CALIN	2	3
312	SY17	Scaffold44	CALIN	4	6
313	SY5	Scaffold20	Complete	2	5
314	SY5	Scaffold27	CALIN	2	2
315	SY5	Scaffold7	CALIN	3	4
316	SY6	Scaffold14	In0	0	1

317	SY6	Scaffold14	CALIN	2	4
318	SY6	Scaffold15	CALIN	3	2
319	SY6	Scaffold5	CALIN	2	2
320	SY9	Scaffold1	CALIN	2	2
321	SY9	Scaffold13	CALIN	2	4
322	SY9	Scaffold13	In0	0	1
323	SY9	Scaffold14	CALIN	3	2
324	SYC	LVDC01000012.1	CALIN	2	4
325	SYC	LVDC01000039.1	CALIN	4	8
326	SYT1	LUCP01000071.1	CALIN	4	6
327	SYT2	LVDT01000012.1	CALIN	2	4
328	SYT2	LVDT01000015.1	Complete	1	5
329	SYT3	LVDW01000035.1	CALIN	2	3
330	SYT3	LVDW01000052.1	In0	0	1
331	SYT3	LVDW01000060.1	CALIN	2	3
332	SYT4	LVDK01000019.1	CALIN	2	1
333	SYT4	LVDK01000022.1	CALIN	2	3
334	TUM17382	AP024614.1	Complete	1	5
335	TUM17383	AP024615.1	CALIN	2	4
336	TUM17386	AP024617.1	In0	0	1
337	TUM4442	AP024610.1	Complete	2	6
338	TUM4442	AP024610.1	CALIN	3	5
339	TYL	LVDS01000055.1	CALIN	2	3
340	VGH117	CP034246.1	In0	0	1
341	VGH117	CP034246.1	CALIN	2	2
342	VGH117	CP034246.1	CALIN	2	2
343	VGH1171	CP032415.1	In0	0	1
344	YHL	LVDU01000004.1	In0	0	1
345	YKSH	DAVXRX010000001.1	Complete	1	3
346	YKSH	DAVXRX010000009.1	CALIN	2	2
347	YTH	LVDA01000021.1	CALIN	3	3
348	YTH	LVDA01000028.1	CALIN	2	2
349	YTL	LVDB01000044.1	CALIN	4	8
350	YZ101	Scaffold32	In0	0	1
351	YZ102	Scaffold32	In0	0	1
352	YZ103	Scaffold28	In0	0	1

Note: In0 elements: they are composed of an integron integrase and no attC sites.
CALIN: attC sites lacking integron-integrases

Table S6. Distribution of antimicrobial resistance genes

Category	Genes	no. of strains
Quinolones	*qnrD1, qnrA1, qnrA2, qnrA3, qnrA6, qnrS4*	206
Sulfonamides	*dfrA1, dfrA27, dfrA36, rsmA, sul1, sul2*	206
β-lactam	bla_{CMY-2}, bla_{NDM-1}, bla_{OXA-4}, bla_{OXA-10}, bla_{VEB-1}, $bla_{TEM-208}$, $bla_{TEM-102}$, bla_{TEM-2}, $bla_{TEM-210}$, bla_{CARB-3}, $bla_{CTX-M-103}$, $bla_{OXA-405}$, $bla_{CTX-M-84}$	206
Aminoglycosides	*aac(3)-Ib, aac(3)-IIb, acrD, ant(3'')-IIa, aph(6)-Id, armA, aadA, aac(3)-IIa, aac(6')-Ib4, ant(2'')-Ia, aadA3, aadA2, aadA16, aadA7, aph(3')-Ia, aph(3'')-Ib, aph(6)-Id, aac(6')-Ip, emrE*	205
Polypeptides	*ugd, mcr*	206
Tetracyclines	*tet(K), tet(A), tet(D), tet(59)*	20
Glycopeptides	*brp(MBL)*	2
Rifamycin	*arr-2*	1
Phenicol	*cmlA5, catI, catB3, floR*	24
Macrolides	*mphA*	3
Associated with multidrug resistance efflux pumps	*crp*	206

Table S7. Mobile genetic elements (MGEs) in the upstream and downstream 10k bp sequences of predicted drug resistance genes

Strain	Drug resistance genes					MGEs		
	Gene	Identity	Scaffold ID	Start	End	Start	End	Function
5	MexD	50.1	Scaffold22	30434	33256	28115	28462	gypsy
6	NmcR	45.2	Scaffold67	121	426	3100	3801	ISb
6	NmcR	45.2	Scaffold67	121	426	5418	6119	ISb
12	MexD	50.1	Scaffold22	30434	33256	28115	28462	gypsy

13	APH(6)-Id	100	Scaffold36	262	1098	3	155	ISb
13	mexM	43	Scaffold31	1019	2641	3104	3589	ISa
13	mexM	43	Scaffold31	1019	2641	5986	7014	ISb
14	qacEdelta1	94	Scaffold17	4838	5380	6444	7637	ISa
A65	AAC(3)-IIb	77.4	JADZGX010000041.1	3812	5452	1744	2466	LINE
ACCC	MexI	77.6	LVCY01000013.1	8840	11914	3999	4481	gypsy
CCU101	TEM-210	100	CP018457.1	66330	67046	70273	71283	ISb
CCU4051	TEM-210	100	CP078514.1	66330	67046	70273	71283	ISb
CCU4052	TEM-210	100	CP078516.1	27798	28514	31741	32751	ISb
CCU4053	NmcR	45.2	CP078517.1	1467	1772	4446	5456	ISb
CCU4054	TEM-210	100	CP078520.1	30018	30734	33961	34971	ISb
CCUG12945	macB	45.2	JADZHV010000018.1	146358	147038	150037	150873	mariner_ant1
CCUG20533	macB	45.2	JADZHT010000031.1	143463	144143	147130	147354	mariner_ant1
CCUG50501	CTX-M-84	53.3	JADZHO010000038.1	19027	19476	16119	16379	gypsy
CCUG526	APH(6)-Id	59	JADZHY010000009.1	81582	84488	80429	80833	mariner_ant1
CCUG72638	MexI	77.5	JADZHL010000017.1	75335	78409	66747	67253	mariner_ant1
CHL	NmcR	45.2	LVDF01000025.1	923	1228	407	628	ISb

CHL	NmcR	45.2	LVDF01000025.1	923	1228	3902	4912	ISb
CLS1	Erm(42)	100	LTBI01000025.1	930	1841	372	797	gypsy
CLS1	Erm(42)	100	LTBI01000025.1	930	1841	2162	3262	ISa
CLS1	Erm(42)	100	LTBI01000025.1	930	1841	8662	8781	mariner
CLS1	MexD	50.1	LTBI01000023.1	22488	25310	20178	20516	gypsy
CLS4	kdpE	55.9	LVDD01000018.1	70542	71261	79225	79797	ISa
CSB04KR	CTX-M-84	60.7	MBFW01000027.1	6927	7847	257	562	gypsy
CSBBB	TEM-102	97	CP047421.1	57085	57318	66354	66806	ISb
CSBBB	pmrE	75.3	CP047422.1	54072	55235	56818	57696	mariner_ant1
DC17SW01	tet(59)	100	JAVMLV010000002.1	199	1401	2446	2667	ISb
DC17SW01	tet(59)	100	JAVMLV010000002.1	199	1401	5941	6447	mariner_ant1
DC17SW02	MexD	50.1	JAVMLU010000013.1	3497	6319	8290	8628	gypsy
DC17SW03	MexD	50.1	JAVMLT010000021.1	3497	6319	8290	8628	gypsy
DC17SW04	MexD	50.1	JAVMLS010000021.1	3497	6319	8290	8628	gypsy
DC17SW05	pmrE	75.8	JAVMLR010000021.1	25052	26215	32925	33431	gypsy
DC17SW06	pmrE	75.8	JAVMLQ010000020.1	25042	26205	32915	33421	gypsy
DC17SW08	pmrE	75.3	JAVMLO010000020.1	2042	3205	5655	6479	mariner
DC18SW01	MexD	50.1	JAVMLM010000041.1	22173	24995	19878	20141	gypsy
DC18SW09	MexD	50.1	JAVMLE010000036.1	5352	8174	10146	10484	gypsy
DC19SW04	TEM-208	72.9	JAVMKZ010000079.1	14	1264	239	817	ISa
HN2022	MexD	50.1	JAODON010000004.1	30434	33256	28136	28366	gypsy

HN2022	NmcR	45.2	JAODON010000039.1	121	426	3100	3801	ISb
HN2022	NmcR	45.2	JAODON010000039.1	121	426	5418	6119	ISb
HT103	macB	45.2	Scaffold10	137630	138310	141309	141923	mariner
HUDD8	OXA-405	61.4	JADZGT010000022.1	72837	73706	66073	66741	ISa
HUDG3	CTX-M-84	55.3	JADZGS010000053.1	422	1339	5791	6651	mariner_ant1
HUDG3	CTX-M-84	55.3	JADZGS010000053.1	422	1339	7216	7788	ISa
HUDG3	macB	45.2	JADZGS010000037.1	143440	144120	147107	147331	mariner_ant1
HUDH4	APH(6)-Id	54.8	JADZGR010000019.1	46527	47435	54722	55228	mariner_ant1
HUDI2	APH(6)-Id	54.8	JADZGQ010000018.1	46527	47435	54722	55228	mariner_ant1
INSAq258	MexI	77.5	JAKCOE010000001.1	8577	11651	34	606	ISa
INSAq495	msbA	45.6	JAKCOV010000004.1	1618	3459	906	1592	mariner_ant1
JCM21037	MexD	50.1	BALO01000011.1	50280	53102	47982	48212	gypsy
JFC3	QnrA3	100	LVCX01000004.1	9344	10000	24	194	mariner_ant1

LC20162	NmcR	45.2	JAGQAR010000096.1	49	354	3028	3729	ISb
LC20162	NmcR	45.2	JAGQAR010000096.1	49	354	5346	6185	ISb
LC20163	NmcR	45.2	JAGQAQ010000098.1	49	354	3028	3729	ISb
LC20163	NmcR	45.2	JAGQAQ010000098.1	49	354	5346	6185	ISb
LZ2013652	pmrE	75.3	JAGQAL010000062.1	5239	6402	7433	7939	gypsy
LZ2015243	MexD	50.1	JAGQAK010000017.1	38608	41430	36307	36636	gypsy
MN01	MexD	49.9	LIRM01000072.1	32320	35142	30010	30348	gypsy
MN01	TEM-208	55.1	LIRM01000019.1	50477	50902	49952	50206	ISa
RC	QnrA3	100	LVCZ01000022.1	9367	10023	2	217	mariner
S12	mphA	69.6	Scaffold32	123	383	5916	6926	ISb
S12	qacEdelta1	100	Scaffold29	117	464	1888	2340	ISb
S159418	APH(6)-Id	59.1	JADZHI010000013.1	8786	11692	89	667	ISa
S16	AAC(3)-IIb	77.4	Scaffold25	7742	9382	5534	6454	LINE
S19	APH(6)-Id	59	Scaffold16	42698	45604	35086	35514	gypsy

S19	APH(6)-Id	65.6	Scaffold36	897	1583	6107	7117	ISb
S19	mphA	100	Scaffold15	134209	135114	142522	142974	ISb
S24	pmrE	75	Scaffold27	59459	60622	61894	62784	mariner_ant1
S26	CTX-M-84	55.3	Scaffold67	2472	3389	4498	5388	mariner_ant1
S28011	TEM-208	54.3	JADZHR010000017.1	4620	5045	4083	4337	ISa
S28011	TEM-208	54.3	JADZHR010000017.1	4620	5045	5875	6129	ISa
S39	MexD	50.1	Scaffold27	2511	5333	237	458	gypsy
S7	mphA	69.6	Scaffold21	123	383	5916	6926	ISb
S7	qacEdelta1	56.7	Scaffold23	508	2049	694	1146	ISb
S97087	AAC(3)-IIb	77.4	JADZHJ010000063.1	3240	4880	1034	1954	LINE
S97087	CTX-M-84	60.7	JADZHJ010000023.1	12494	13306	13420	14244	mariner
SY101	MexD	50.1	Scaffold22	24608	27430	22298	22636	gypsy
SYC	FosA	42.3	LVDC01000027.1	7567	8466	59	781	ISa
TYL	MexD	50.1	LVDS01000045.1	22274	25096	19964	20302	gypsy
VGH1171	NmcR	45.2	CP032414.1	1467	1772	4446	5456	ISb

YTH	QnrA3	100	LVDA01000023.1	9352	10008	2	202	mariner

Table S8. The results of antimicrobial resistance genes analysis of 62 *S. algae* strains

Strain	TCC	TZP	CAZ	CPS	FEP	ATM	IPM	MEM	AMK	TM	CIP	LEV	DO	MNO	TGC	CS	SXT
6638	S	S	S	S	S	R	S	S	S	S	S	S	S	S	S	S	S
6	S	S	S	S	S	S	S	S	S	S	R	I	S	S	S	R	R
5	S	S	S	S	S	S	R	S	S	S	R	S	S	S	S	S	S
12	S	S	S	S	S	S	R	S	S	S	R	S	S	S	S	S	S
13	S	I	S	S	I	S	S	S	S	R	S	S	S	S	S	R	R
14	S	S	S	S	S	S	R	S	S	I	S	S	R	S	S	R	R
S1	S	S	S	S	S	S	S	S	S	S	R	S	S	S	S	S	S
S2	S	S	S	S	S	S	R	S	S	S	S	S	S	S	S	R	S
S3	S	S	S	S	S	S	R	S	S	S	S	S	S	S	S	R	S
S4	S	S	S	S	S	S	R	S	S	S	S	S	S	S	S	R	S
S5	S	S	S	S	S	S	R	S	S	S	S	S	S	S	S	R	S
S6	S	S	S	S	S	S	R	S	S	S	S	S	S	S	S	S	S
S7	R	R	R	R	I	S	R	R	S	R	S	S	S	S	S	R	R
S8	S	S	S	S	S	S	S	S	S	S	S	S	S	S	S	R	S
S9	S	S	S	S	S	S	S	S	S	S	S	S	S	S	S	R	S
S10	S	S	S	S	S	S	S	S	S	S	S	S	S	S	S	R	S
S11	S	S	S	S	S	S	S	S	S	S	S	S	S	S	S	R	S

S12	R	R	R	R	I	S	R	R	S	I	S	S	S	S	S	S	S
S13	S	S	S	S	S	S	R	S	S	S	S	S	S	S	S	R	S
S14	S	S	S	S	S	S	R	S	S	S	S	S	S	S	S	R	S
S15	S	S	S	S	S	S	R	S	S	S	S	S	S	S	S	R	S
S16	S	S	S	S	S	S	R	S	S	S	S	S	S	S	S	S	S
S17	S	S	S	S	S	S	R	S	S	S	S	S	S	S	S	R	S
S18	S	S	S	S	S	S	S	S	S	S	S	S	S	S	S	S	S
S19	S	S	S	S	S	S	S	S	S	S	S	S	S	S	S	S	R
S20	S	S	S	S	S	S	S	S	S	S	S	S	S	S	S	S	S
S21	S	S	S	S	S	S	R	S	S	S	S	S	S	S	S	S	S
S22	S	S	S	S	S	S	R	S	S	S	S	S	S	S	S	R	S
S24	S	S	S	S	S	S	S	S	S	S	S	S	S	S	S	R	S
S26	S	S	S	S	S	S	S	S	S	S	S	S	S	S	S	S	S
S27	S	S	S	S	S	S	S	S	S	S	S	S	S	S	S	S	S
S28	S	S	S	S	S	S	S	S	S	S	S	S	S	S	S	R	S
S31	S	S	S	S	S	S	R	S	S	S	S	S	S	S	S	S	S
S32	S	S	S	S	S	S	S	S	S	S	S	S	S	S	S	S	S
S33	S	S	S	S	S	S	R	S	S	S	I	S	S	S	S	R	S
S34	S	S	S	S	S	S	R	S	S	S	S	S	S	S	S	R	S
S35	S	S	S	S	S	S	R	S	S	S	S	S	S	S	S	R	S
S36	S	S	S	S	S	S	S	S	S	S	S	S	S	S	S	S	S
S37	S	S	S	S	S	S	R	S	S	S	S	S	S	S	S	S	S
S39	S	S	S	S	S	S	R	S	S	S	S	S	S	S	S	R	S
S40	S	S	S	S	S	S	S	S	S	S	S	S	S	S	S	S	S
S41	S	S	S	S	S	S	S	S	S	S	S	S	S	S	S	S	S

S42	S	S	S	S	S	S	S	S	S	S	S	S	S	S	S	S	S
S43	S	S	S	S	S	S	S	S	S	S	S	S	S	S	S	R	S
SY1	S	S	S	S	S	S	S	S	S	S	S	S	S	S	S	S	S
SY4	S	S	S	S	S	S	S	S	S	S	S	S	S	S	S	S	S
SY5	S	S	S	S	S	S	R	S	S	S	S	S	S	S	S	S	S
SY6	S	S	S	S	S	S	R	S	S	S	S	S	S	S	S	S	S
SY9	S	S	S	S	S	S	R	S	S	S	S	S	S	S	S	S	S
SY11	S	S	S	S	S	S	S	S	S	S	S	S	S	S	S	R	S
SY15	S	S	S	S	S	S	S	S	S	S	S	S	S	S	S	S	S
SY17	S	S	S	S	S	S	S	S	S	S	S	S	S	S	S	S	S
SY101	S	S	S	S	S	S	S	S	S	S	S	S	S	S	S	R	S
SY102	S	S	S	S	S	S	S	S	S	S	S	S	S	S	S	R	S
SY103	S	S	S	S	S	S	S	S	S	S	S	S	S	S	S	S	S
SY104	S	S	S	S	S	S	R	S	S	S	S	S	S	S	S	S	S
HT103	S	S	S	S	S	S	S	S	S	S	S	S	S	S	S	S	S
TH101	S	S	S	S	S	S	R	S	S	S	S	S	S	S	S	S	S
XD101	S	S	S	S	S	S	I	S	S	S	S	S	S	S	S	S	S
YZ101	S	S	S	S	S	S	R	S	S	S	S	S	S	S	S	S	S
YZ102	S	S	S	S	S	S	S	S	S	S	S	S	S	S	S	S	S
YZ103	S	S	S	S	S	S	R	S	S	S	S	S	S	S	S	S	S

Note: TCC: Ticarcillin/Clavulanic acid; TZP: Piperacillin/Tazobactam; CAZ: Ceftazidime; CPS: Cefoperazone/Sulbactam; FEP: Cefepime; ATM: Aztreonam; IPM: Imipenem; MEM: Meropenem; AMK: Amikacin; TM: Tobramycin; CIP: Ciprofloxacin; LEV: Levofloxacin; DO: Doxycycline; MNO: Minocycline; TGC: Tigecycline; CS: Colistin; SXT: Trimethoprim/Sulfamethoxazole. Pseudomonas aeruginosa ATCC 27853 was used as a control.

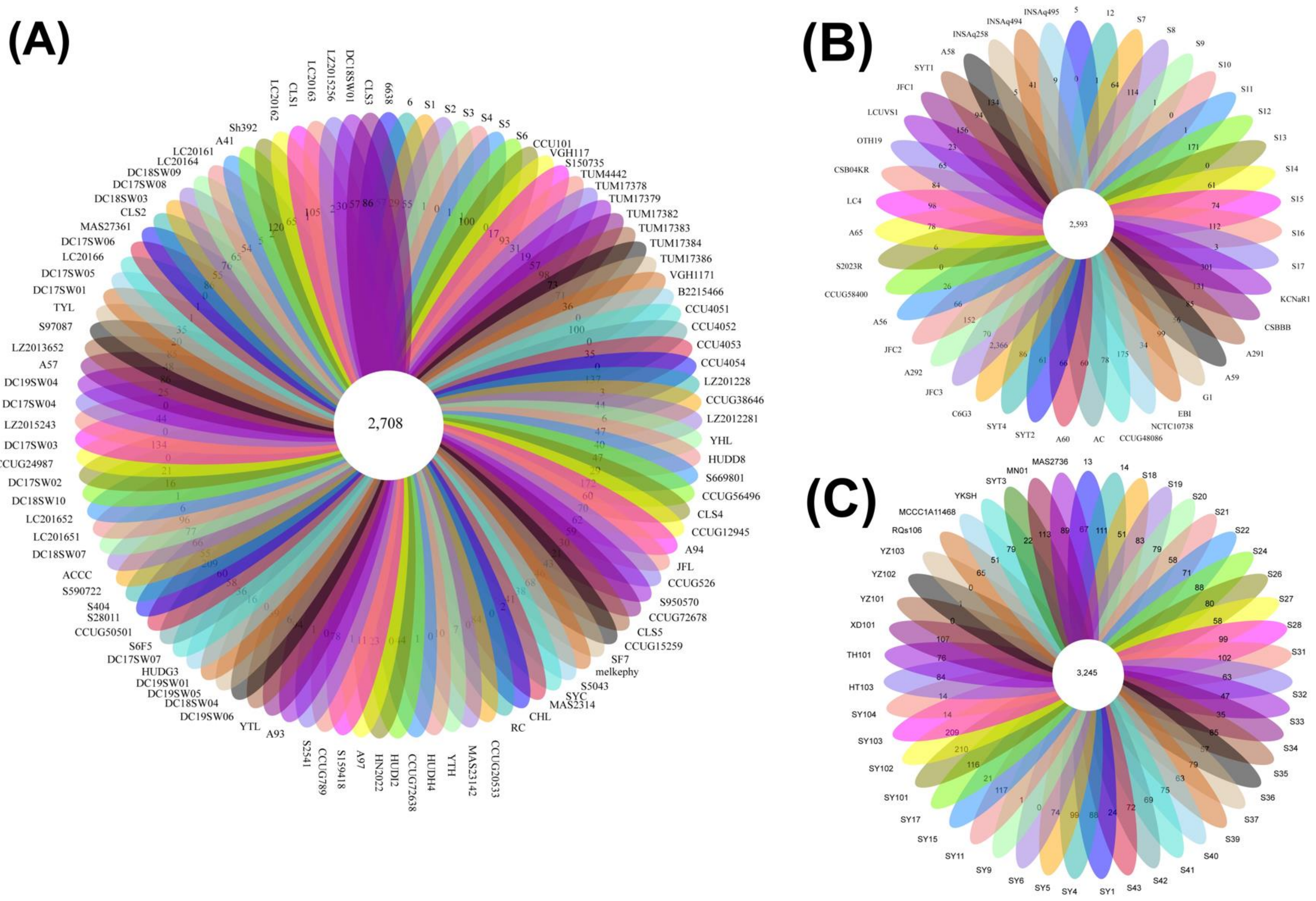

Figure S1. The distribution of unique genes among clinical, animal, and environmental strains. (a) The unique genes distribution of clinical strains; (b) The unique genes distribution of animal strains; (c) The unique genes distribution of environmental strains.

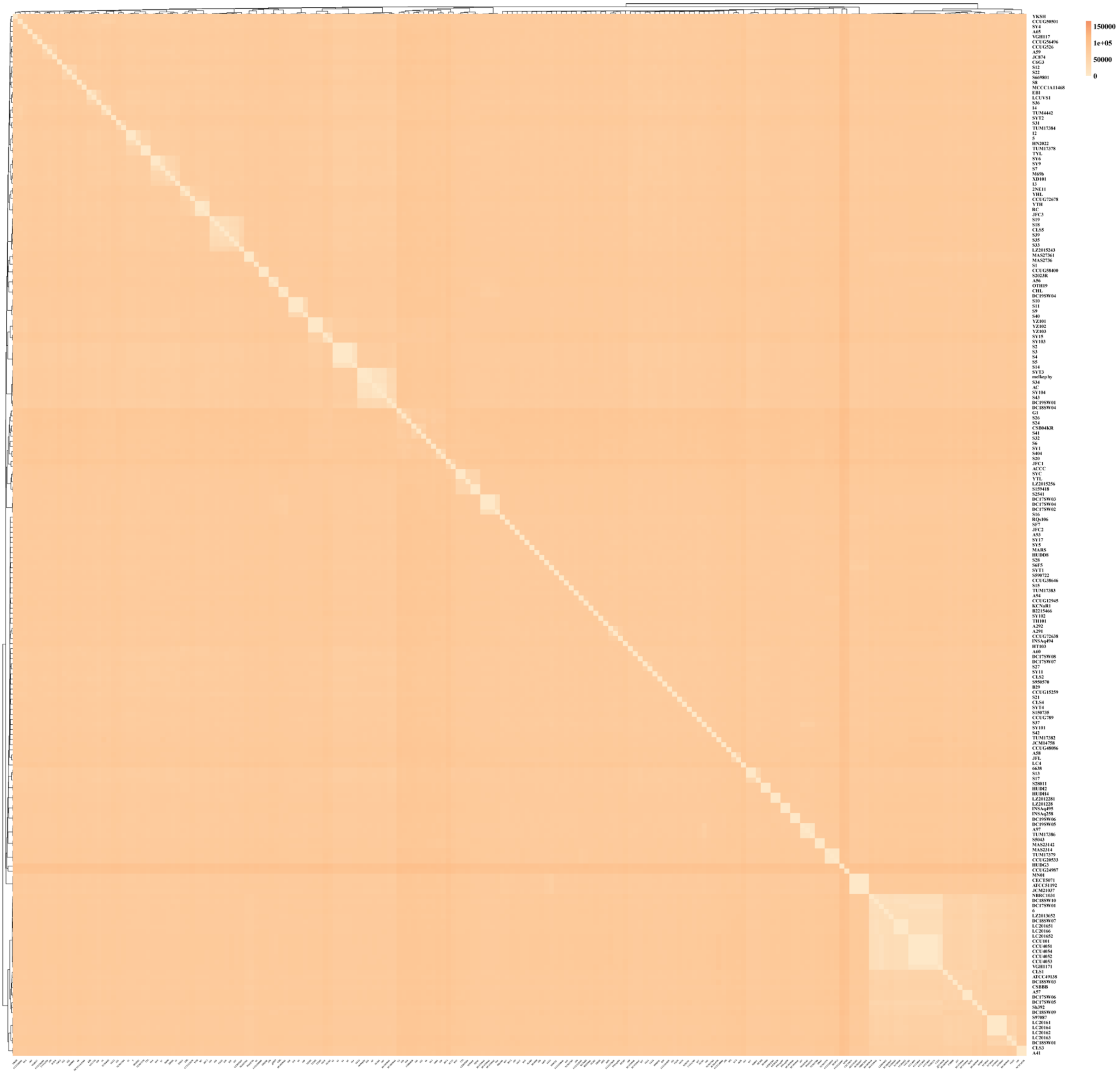

Figure S2. The distribution of pairwise SNP distances of 206 *S. algae* strains.

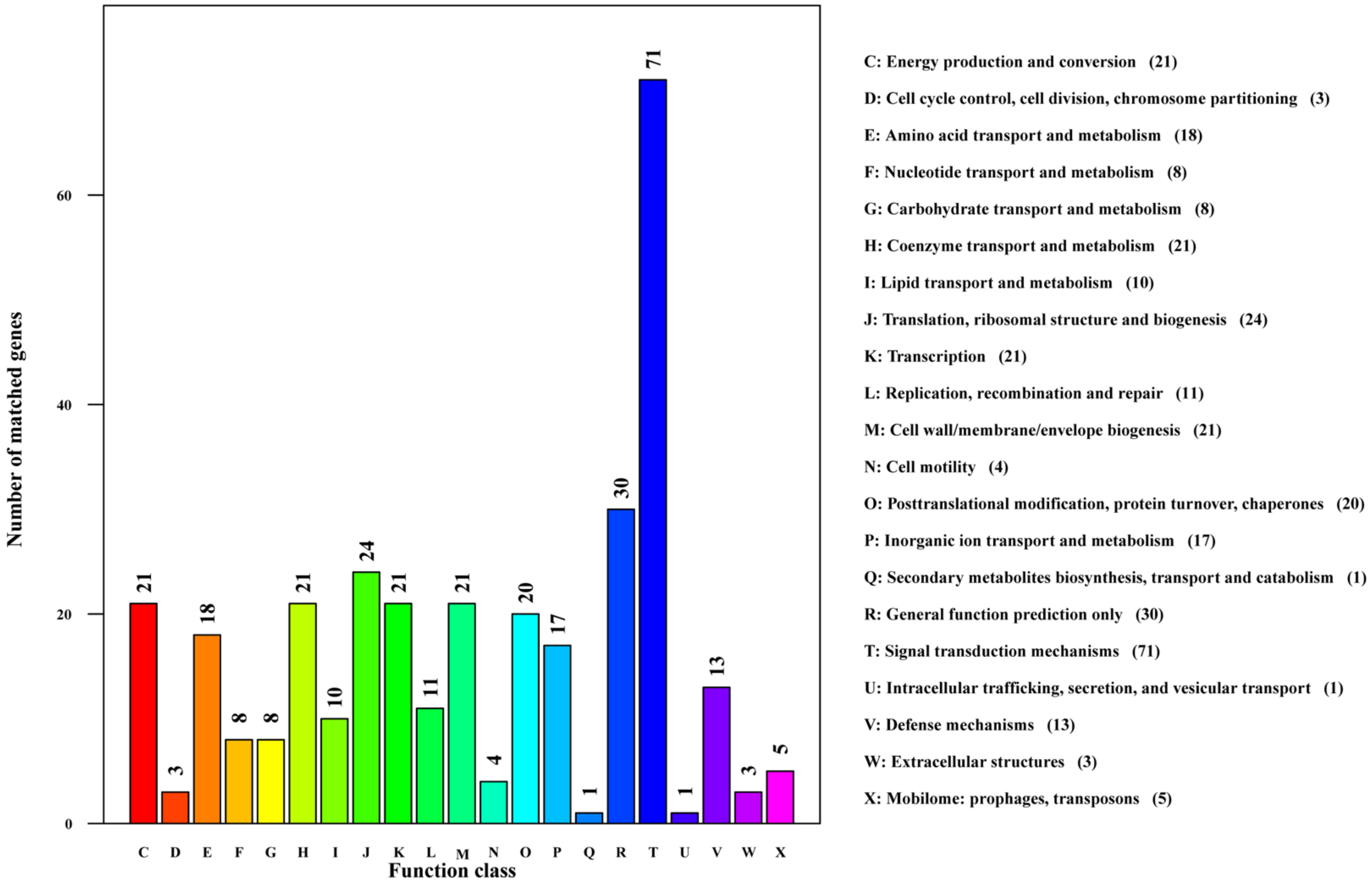

Figure S3. The COG function classification of the 206 strains unique genome
